# HBV maintains electrostatic homeostasis by modulating negative charges from phosphoserine and encapsidated nucleic acids

**DOI:** 10.1038/srep38959

**Published:** 2016-12-13

**Authors:** Pei-Yi Su, Ching-Jen Yang, Tien-Hua Chu, Chih-Hsu Chang, Chiayn Chiang, Fan-Mei Tang, Chih-Yin Lee, Chiaho Shih

**Affiliations:** 1 Taiwan International Graduate Program in Molecular Medicine, National Yang-Ming University and Academia Sinica, Taipei, Taiwan; 2 Institute of Biomedical Sciences, Academia Sinica, Taipei, Taiwan; 3 Institute of Biochemistry and Molecular Biology, National Yang-Ming University, Taipei, Taiwan; 4 National Defense Medical Center, Taipei, Taiwan; 5 Graduate Institute of Microbiology, College of Medicine, National Taiwan University, Taipei, Taiwan

## Abstract

Capsid assembly and stability of hepatitis B virus (HBV) core protein (HBc) particles depend on balanced electrostatic interactions between encapsidated nucleic acids and an arginine-rich domain (ARD) of HBc in the capsid interior. Arginine-deficient ARD mutants preferentially encapsidated spliced viral RNA and shorter DNA, which can be fully or partially rescued by reducing the negative charges from acidic residues or serine phosphorylation of HBc, dose-dependently. Similarly, empty capsids without RNA encapsidation can be generated by ARD hyper-phosphorylation in insect, bacteria, and human hepatocytes. De-phosphorylation of empty capsids by phosphatase induced capsid disassembly. Empty capsids can convert into RNA-containing capsids by increasing HBc serine de-phosphorylation. In an HBV replicon system, we observed a reciprocal relationship between viral and non-viral RNA encapsidation, suggesting both non-viral RNA and serine-phosphorylation could serve as a charge balance buffer in maintaining electrostatic homeostasis. In addition, by comparing the biochemistry assay results between a replicon and a non-replicon system, we observed a correlation between HBc de-phosphorylation and viral replication. Balanced electrostatic interactions may be important to other icosahedral particles in nature.

Hepatitis B Virus (HBV) is a severe public health problem globally^1^,^2^. Two billion people have been infected with HBV worldwide, and to date, there is still no effective treatment to eradicate the virus in chronic carriers^3^. HBV replicates by reverse transcription via a pregenomic RNA (pgRNA) intermediate^4^,^5^. In addition to the full-length 3.5 kb pgRNA, HBV can generate spliced RNAs^6^,^7^,^8^, which in turn can be reverse transcribed into spliced DNAs in cell culture and patients^9^,^10^,^11^.

HBV contains four major open reading frames (ORFs), which encode precore/core, surface (envelope), polymerase, and X proteins. The core protein (HBc) contains 183–185 amino acids and can self-assemble into icosahedral capsid particles (capsids)^12^,^13^,^14^,^15^,^16^. Polypeptide HBc149–183 at the carboxyl-terminus is an arginine-rich domain (ARD), which contains a total of 16 clustering arginines and can be divided into four subdomains (ARD-I, -II, -III, and -IV) (Fig. 1A). HBc ARD can bind with nucleic acids^14^,^17^,^18^,^19^, and is important for intracellular trafficking of HBc^20^,^21^,^22^,^23^,^24^. Recently, ARD was shown to contain nucleic acid chaperon activity^25^, antimicrobial activity^26^,^27^ and is required for secretion of empty virions^28^.

Both duck hepatitis B virus (DHBV) core protein and human HBc are known to be phosphorylated *in vivo*^29^,^30^,^31^,^32^,^33^,^34^. In HBV, three of the seven serines are major phosphorylation sites^34^ (Fig. 1A). Serine phosphorylation and de-phosphorylation of HBc play an important role in viral RNA encapsidation and DNA synthesis^33^,^35^,^36^,^37^,^38^,^39^,^40^,^41^. It remains to be elucidated how HBc phosphorylation could have an effect on many diverse biological activities of HBV. Since phosphorylation at the serine residues of HBc ARD could alter the negative charge content in the capsid interior, it is natural to ask whether serine phosphorylation could affect charge balance, leading to altered capsid stability, assembly, RNA encapsidation, and DNA synthesis.

HBV exists in patients as a highly heterogeneous population. The mechanism for the generation of such a high degree of diversity in HBV particles remains to be elucidated. For example, it remains unclear why in the sera of hepatitis B patients, empty virions without any packaged DNA or RNA are highly abundant^42^,^43^,^44^. Yet, in other cases, HBV particles containing spliced viral genome have been reported^6^,^11^. This phenomenon has also been documented in the human cell culture system^7^,^45^,^46^. When HBc was expressed in the *baculovirus* system, empty capsid particles (empty capsids) were also observed^47^.

Previously, we proposed a charge balance hypothesis which highlights the importance of balanced electrostatic interactions between the positive charge from HBc ARD and the negative charge from encapsidated nucleic acids and HBc itself^40^,^48^,^49^. Such electrostatic interactions in the capsid interior could affect viral capsid stability, assembly, RNA encapsidation and DNA replication (Fig. 1B). We tested *in vitro* this hypothesis by using a capsid disassembly and reassembly system, as well as *in vivo* by using a charge rebalance approach in a truncated HBc context^48^,^49^. Despite these preliminary studies, the working hypothesis remains highly speculative, since there are many important issues that need to be addressed: (1) If charge balance is indeed so important, how can it explain the formation of empty capsids without any encapsidated RNA in natural infection and cell culture^42^,^43^,^44^,^45^,^46^? (2) Does encapsidation of non-viral or cellular RNA, if any, play any role in the electrostatic interaction in HBV capsids? (3) As mentioned above, HBc ARD contains a large number of serine phosphorylation sites. Is it possible that phosphoserine could contribute to balance electrostatic interactions? If so, are they functionally interchangeable and substitutable to encapsidated RNA, DNA, or acidic residues of HBc? (4) In addition to a genetic approach via phospho-mimicking mutagenesis, can a more direct biochemistry approach be used in the studies of the potential involvement of phosphorylation in electrostatic homeostasis? (5) In our earlier experiments using a truncated mutant HBc contex^40^,^48^, it remains unclear if the successful rescue in viral replication is due to the increased length of the cytoplasmic tail of HBc, or due to the increased arginine content in the ARD domain of HBc^40^? Can the charge rebalance experiment be performed in a more natural full-length context^48^?

In this study, for the first time, we demonstrated a good correlation between the arginine content of HBc ARD in the full-length HBc context and the size of encapsidated viral RNAs and DNAs. Arginine-deficient mutants encapsidated shorter RNA and DNA than the wild type (WT) HBV. By DNA sequencing, we discovered a number of novel spliced viral RNA species encapsidated in arginine-deficient ARD mutants. These mini-RNA species are not abundant and could not have been found in wild type HBV populations. They become concentrated and enriched only in a capsid environment in severe shortage of positive charge, since they are the fittest match selected by the charge balance principle.

By reducing negative charges at acidic residues or serine phosphorylation sites of HBc, we can restore fully or partially the shorter RNA and DNA phenotype of arginine-deficient ARD mutants. Therefore, it appears that negative charge involved in balanced electrostatic interactions in the capsid interior can be donated from three interchangeable or substitutable sources, including encapsidated RNA, acidic residues of HBc, and serine phosphorylation at HBc ARD. Interestingly, using an HBc expression vector for VLP (virus-like particle) production, we demonstrated for the first time that hyper-phosphorylation of HBc is responsible for the formation of empty capsids in insect, *E. coli*, and human hepatocytes. Furthermore, by using an HBV replicon system, we observed an inverse correlation between the amounts of encapsidated viral and non-viral RNAs. This result suggests that the encapsidated non-viral RNA might function as a charge balance buffer to help maintain an optimum ratio between the positive and negative charges in the capsid interior.

In summary, balanced electrostatic interactions appear to be a ground rule in guiding the behaviors of HBV in its life cycle. Balanced electrostatic interaction in the capsid interior is dependent upon (1) positively-charged residues of the core protein; (2) negative charges of acidic or phosphorylated residues; and (3) negative charges of the encapsidated total RNA/DNA contents, controlled by splicing of viral RNA or the co-encapsidation of cellular RNA. Our current studies on capsid assembly/disassembly could provide useful information for translational research in anti-virals, VLP-based vaccines, and drug delivery via nanoparticles or liposomes.

## Results

### The size of encapsidated viral DNA and RNA is determined by the positive charge content at HBc ARD

**Figure 1:**
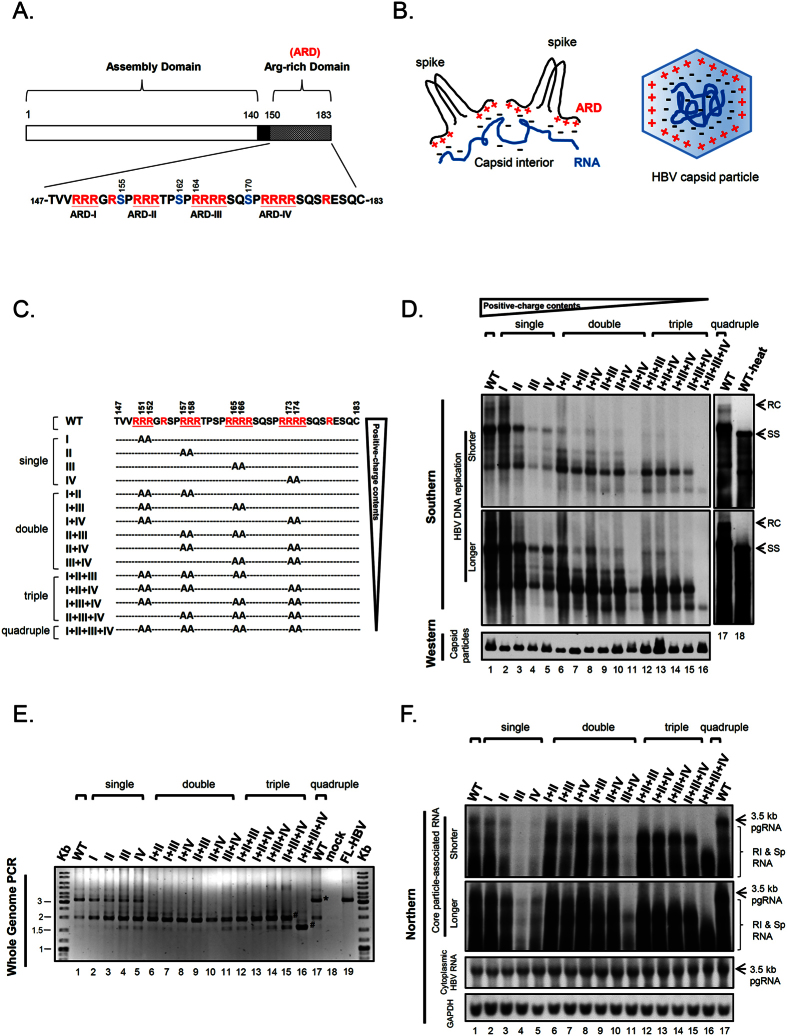
Gradual reduction in the positive charge contents of HBc correlated with the gradual reduction in the size and amount of viral particle-associated HBV DNA and RNA. (**A**) HBc consists of two distinct domains responsible for capsid assembly and viral RNA encapsidation. Three major phosphorylation sites at serine (S) 155, 162 and 170 are shown in blue^34^. Arginines (R) are shown in red. (**B**) The charge balance hypothesis (electrostatic homeostasis) highlights the electrostatic interactions between the positive charge from HBc arginine-rich domains (ARD) and the negative charge from encapsidated nucleic acids. Such a balanced or imbalanced electrostatic interaction in the capsid interior could regulate viral RNA encapsidation, DNA replication, capsid conformation, stability and assembly^40^,^48^,^49^. (**C**) Construction of 15 different ARD mutants with different R-to-A substitutions at different positions. Viral DNA and RNA from transfected culture were analyzed. (**D**) *Upper panel:* Both numbers and positions of positive-charged arginine could strongly influence the size and intensity of HBV DNA replicative intermediates by Southern blot analysis. RC: full-length relaxed circle DNA (4.0 kb). Heat-denatured HBV DNAs (100 °C, 5 min) banded as smearing signals starting from the 1.5 kb SS (single-strand) DNA position (lane 18). Smearing signals represent HBV DNA replicative intermediates with different MW. *Lower panel*: Intracellular capsid particles were assayed by native agarose gel electrophoresis and Western blot using an anti-HBc antibody. (**E**) The core particle-associated DNAs were analyzed by Whole Genome PCR (M&M^10^) and agarose gel electrophoresis with ethidium bromide staining. The mainstream population of encapsidated viral DNAs decreased in size when the arginine content was decreased. #Shorter form HBV DNA; *Full-length 3.2 kb HBV DNA (lane 17); FL-HBV: 3.2 kb full-length DNA amplified from an HBV plasmid (lane 19). (F) *Upper panel*: HBV core-associated viral RNAs were analyzed by Northern blot. Consistent with the gradual reduction in viral DNA sizes in (**D**,**E**), the size reduction of encapsidated RNA was associated with the gradual reduction of the positive charge content of HBc ARD. RI and Sp RNA: replicative intermediates of reverse-transcribing viral RNAs and spliced RNAs. *Lower panel*: total cytoplasmic HBV RNA and GAPDH were included as controls.

Previously, we characterized an HBc ARD mutant 164 which truncates 19 amino acids from the C-terminus of the full-length HBc183 (Fig. 1A). This mutant 164 encapsidated predominantly short viral DNAs^40^. When the C-terminus of the truncated HBc164 was progressively extended in length, the encapsidated DNAs progressively increased in both size and signal intensities. Since the 19 amino acids truncated in mutant 164 includes a total of 8 arginines, the short DNA phenotype of mutant 164 could result from either the shortened length or the reduced arginine content of HBc ARD. To differentiate between these two possibilities, we constructed a total of 15 different ARD mutants with varying arginine contents in the same full-length HBc context via arginine-to-alanine (R-to-A) substitutions (Fig. 1C). HBV replicon plasmids containing these HBc arginine-deficiencies were transfected into human hepatoma HuH-7 cells, and intracellular viral DNAs were analyzed by Southern blot (Fig. 1D). As expected^25^,^50^, DNA replication of mutants ARD-III and ARD-IV was dramatically reduced relative to those of mutants ARD-I and ARD-II. While the full-length relaxed circle (RC) DNA form remained detectable in mutant ARD-I (lane 2, Fig. 1D), it was not visually detectable in the other three single ARD mutants (lane 3–5, Fig. 1D; see further discussions below). The importance of domains ARD-III and ARD-IV is also evident in the double, triple and quadruple mutants. Interestingly, viral DNA replication of double, triple, and quadruple ARD mutants reveals a further downshift pattern of HBV DNA, indicating that the molecular weights of replicative intermediates are progressively decreasing, in parallel with the progressive reduction in the positive charge contents of ARD. These ARD mutants remained capable of capsid assembly as measured by native agarose gel and Western blot analysis (*lower panel* of Fig. 1D). While the full-length RC form of most ARD mutants were not easily detectable by Southern blot (Fig. 1D), it can be clearly detected in some single ARD mutants after extra-longer exposure (lane 3 and 5, Supplementary Figure S1A) or by the more sensitive Whole Genome PCR analysis (lane 3–5, Fig. 1E). Full-length RC DNA can be amplified by Whole Genome PCR to produce a 3.2 kb DNA fragment (M&M^10^). As shown in lane 1 and 17 (Fig. 1E), wild type HBV exhibited one major band around 3.2 kb and a minor band around 2 kb. The 3.2 kb band co-migrated with a full-length HBV (FL-HBV) DNA size marker PCR amplified from an HBV plasmid DNA template (lane 19). The minor band around 2 kb represented a predominant population of “short form” viral DNA in lanes 2–15. When encapsidated HBV DNAs of single ARD mutants were analyzed by the Whole Genome PCR, both 3.2 kb and 2 kb DNA remained detectable (lane 2–5). However, this 3.2 kb full-length DNA species were not detectable in double, triple, and quadruple ARD mutants (lane 6–16). It is noteworthy that the quadruple ARD mutant lost both 3.2 kb and 2 kb DNA species, yet displayed an intense banding at the 1.5 kb position, which was also detectable as a faint banding in the other ARD mutants and WT (lane 1–15, Fig. 1E). Overall, in this qualitative PCR assay, the most predominant population of encapsidated viral DNAs appeared to gradually downshift in size from 3.2 kb to 2 kb and finally to 1.5 kb. This trend seems to be in parallel to the gradient of the decreasing positive charge content in ARD mutants.

As a DNA virus, HBV replicates via reverse transcription^5^. The “short DNA phenotype” of ARD mutants in Fig. 1D,E could result from aberrant encapsidation of a short RNA species. Alternatively, it could reflect a defect in reverse transcription of the normal-sized 3.5 kb RNA pregenome. To differentiate between these two possibilities, we analyzed the core-associated viral RNAs from ARD mutants by Northern blot analysis (Fig. 1F). The result indicates that ARD mutants with a reducing number of arginines exhibited a trend to package an increasing amount of shorter-sized RNA species. The quadruple mutant ARD-I + II + III + IV encapsidated the shortest species of viral RNA (lane 16, Fig. 1F), which can then be reverse transcribed into the shortest DNA species (lane 16, Fig. 1D,E). Similarly, the strong phenotypic defect in DNA synthesis of mutants ARD-III, ARD-IV, and ARD-III + IV originated from their upstream defect in viral RNA encapsidation (Fig. 1F and Supplementary Figure S1B).

### The short DNA phenotype of ARD mutants originates from encapsidation of spliced RNAs

**Figure 2:**
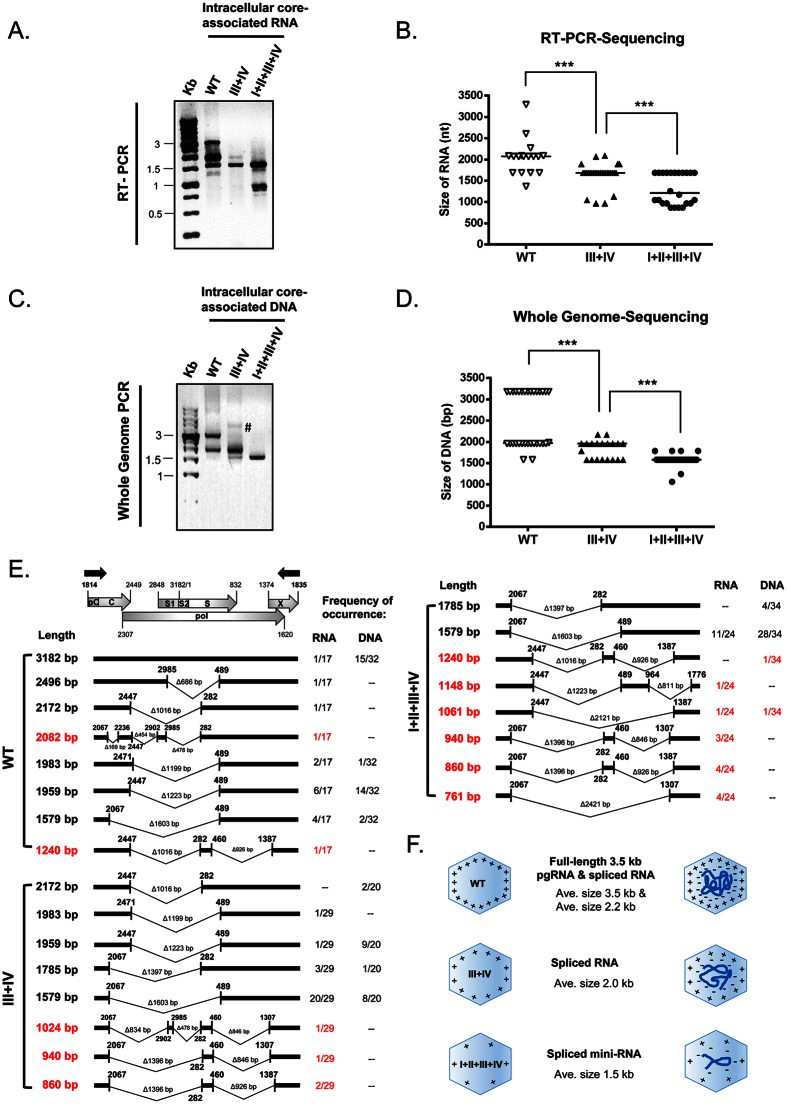
Arginine-deficient HBc mutants preferentially encapsidated spliced viral RNA and DNA. **(A)** Core particle -associated viral RNAs of HBc ARD mutants were analyzed by RT-PCR and agarose gel with ethidium bromide staining. The positions of the RT-PCR primers are shown as black arrows in Fig. 2E. Compared to WT, mutant ARD-III + IV encapsidated shorter-sized viral RNAs, while mutant ARD-I + II + III + IV encapsidated the shortest RNA species. **(B)** The diagram summarized the statistics of RT-PCR sequencing results of core particle -associated viral RNAs of WT, mutants ARD-III + IV and I + II + III + IV. Each symbol represents one independently isolated clone from *E. coli*. The horizontal line represents the medium size of encapsidated RNA species. ***p < 0.001, by Student’s *t*-test. **(C)** Core particle-associated viral DNAs of HBc ARD mutants were analyzed by Whole Genome PCR and agarose gel with ethidium bromide staining. The positions of the Whole Genome PCR primers are near the triple-stranded DNA region (M&M^10^). Compared to WT, mutant ARD-III + IV encapsidated shorter -sized viral DNA, while mutant ARD-I + II + III + IV encapsidated the shortest-sized DNA. Symbol # marked a faint higher MW band which was not detected in other repeat experiments. **(D)** The diagram showed the statistics of PCR sequencing results of core particle-associated viral DNAs of WT, mutants ARD-III + IV and I + II + III + IV. Each symbol represents one independently isolated clone from *E. coli*. The horizontal line represents the medium size of encapsidated DNA species. ***p < 0.001, by Student’s *t*-test. **(E)** Sequencing results and frequencies of occurrence of each spliced species in *E. coli* clones from wild type and ARD mutants. A schematic representation of HBV pgRNA includes the pC (pre-core), C (core), pol (polymerase), S1/S2/S (surface proteins) and X (X protein). The red color represents novel spliced transcripts not reported previously in literature. **(F)** The cartoon highlights the trend of encapsidating smaller-sized RNA and DNA, when the arginine content at HBc ARD is reduced.

To identify these short RNA species encapsidated in ARD mutants, we performed RT-PCR sequencing (Fig. 2A,B) and Whole Genome DNA Sequencing (Fig. 2C,D) of intracellular core particle-associated viral RNAs and DNAs. Interestingly, in addition to the known spliced RNA species^7^,^9^,^11^,^51^,^52^,^53^,^54^,^55^,^56^, we identified 8 novel species of spliced mini-RNA encapsidated in arginine-deficient mutants III + IV and I + II + III + IV (red color in Fig. 2E). Except for two novel RNA species 1240 nt and 1061 nt, none of the rest of these novel RNAs can be detected to have their reverse transcribed cDNA counterparts by Whole Genome DNA analysis. Furthermore, only one novel spliced DNA species (940 bp) was detected in extracellular viral particles of mutant ARD- I + II + III + IV (Supplementary Figure S2; see Discussion). Our results indicate that the arginine-deficient ARD mutants (with less positive charge), preferentially encapsidated spliced RNA (with reduced negative charge), over the full-length pgRNA (with “excessive” amount of negative charge). Overall, ARD mutants with progressively reduced number of arginines tend to encapsidate progressively shorter and shorter RNA and DNA species in both intracellular and extracellular viral particles. In contrast, when the amount of RNA and arginine content are not properly matched with each other, stable capsids cannot be formed, since excessive amounts of negative or positive charge in the capsid interior lead to charge repulsion and capsid disassembly (Fig. 2F). As a side note, in Fig. 2E, although we can PCR amplify HBV DNA template with a good efficiency (frequency of occurrence15/32), we cannot RT-PCR amplify the full-length 3.2 kb RNA template at a high efficiency (1/17). This is likely due to the notorious inefficiency of reverse transcription *in vitro*. We noted that WT HBV also encapsidated a highly abundant 2.2 kb spliced RNA (1959 nt in Fig. 2E; See Discussion).

### Negatively-charged acidic residues in the full-length HBc context contribute to charge balance

**Figure 3:**
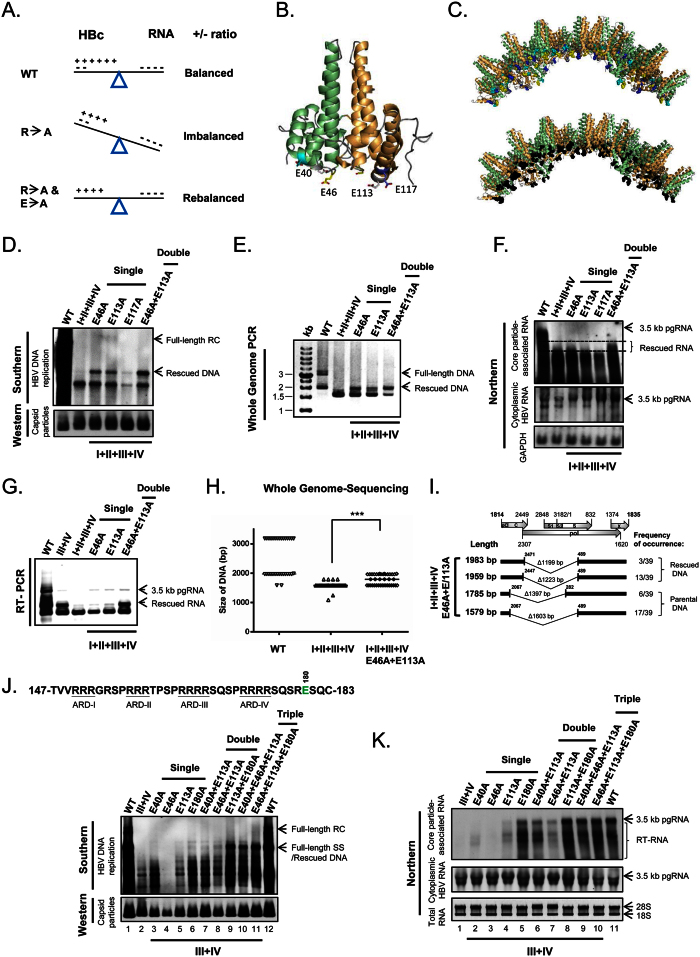
A charge-rebalance approach via reducing acidic residues of HBc. **(A)** A cartoon illustrates the charge rebalance approach. **(B)** The dimeric structure of HBc (PDB ID:1QGT) with each monomer in green and orange ribbon modes. The side chains of four glutamic acids are located close to the capsid interior: E40 (cyan), E46 (yellow), E113 (white) and E117 (blue)^18^,^70^,^71^,^90^. **(C)** The schematic presentation of a 40-mer HBc in the context of a 240-mer icosahedral particle (T = 4) is illustrated using PyMol (DeLano Scientific LLC, Palo Alto, CA, USA). *Upper panel*: E40 (cyan), E46 (yellow), E113 (white) and E117 (blue). *Lower panel*: all four acidic residues are in black. **(D)** Parental HBc mutant ARD-I + II + III + IV exhibited a very short DNA phenotype by Southern blot, which can be partially rescued by E-to-A single or double mutations at E46 or E113. **(E)** The Southern result in (**D**) can be confirmed by Whole Genome PCR analysis using core particle-associated viral DNAs as a template. **(F)** *Upper panel*: Northern blot analysis of HBc parental mutant ARD-I + II + III + IV and its charge rebalanced derivatives. Rescued viral RNAs with significantly increased size (between two dotted lines) were observed in HBc mutant E46A + E113A (last lane). *Lower panels*: Total cytoplasmic HBV RNA and GAPDH RNA were included as controls. **(G)** The Northern results in (**F**) was confirmed by RT-PCR analysis using core particle-associated viral RNAs. **(H)** Size distributions of encapsidated viral DNAs in WT, mutant ARD-I + II + III + IV, and its double-rescued mutant. Each clone was characterized by sequencing as described in Fig. 2D. The horizontal line represents the medium size of encapsidated viral DNA species. ***p < 0.001, Student’s *t*-test. **(I)** Novel spliced transcripts (1983 bp and 1959 bp) can be rescued in mutant ARD-I + II + III + IV containing E46A and E113A mutations. **(J)** *Upper panel:* The acidic residue E180 (green) is located in the ARD without a known structure^18^,^57^. *Lower panel:* Defective DNA replication of mutant ARD-III + IV can be synergistically rescued by multiple E-to-A mutations in a dose-dependent manner using Southern blot analysis. **(K)** The encapsidated RNA of mutant ARD-III + IV can be efficiently rescued in a dose-dependent manner by multiple charge rebalance mutations using Northern blot analysis.

Since arginines at HBc ARD can contribute positive charge to the balanced electrostatic interactions, we asked if, in addition to RNA/DNA, the HBc protein itself can contribute any negative charge to electrostatic homeostasis in the capsid interior? To address this possibility, we used a charge rebalance approach. In the *top panel* of Fig. 3A, wild type HBc and its encapsidated RNA are at a charge balanced status. In the *middle panel*, charge imbalance is caused by arginine-deficiency. In the l*ower panel*, charge imbalance may be remedied via a secondary E-to-A mutation of HBc. To test this charge rebalance strategy in the full-length HBc context, we need to know which acidic residues on HBc capsid particles are more likely to be involved in balancing electrostatic interactions. Using the PyMol software, we selected four acidic residues E40, E46, E113, and E117 for testing. These residues are located near the inner surface of the protein shell, and except for E40, their acidic side chains clearly point into the capsid interior (Fig. 3B,C).

This charge rebalance approach was tested using various arginine-deficient contexts of different ARD mutants, including ARD-III, ARD-IV, ARD-III + IV, and ARD-I + II + III + IV (Fig. 1C). The rescue results from these different rebalance experiments using different ARD mutants shared a similar trend to generate longer RNA or DNA products, and we present here only examples from ARD-III, ARD-IV (Supplementary Figure S3), ARD-III + IV (Fig. 3J,K), and ARD-I + II + III + IV (Fig. 3D–I). Since mutant ARD-I + II + III + IV exhibited a very short DNA phenotype (Figs 1 and 2), we asked whether this phenotype can be rescued by charge rebalanced E-to-A mutations at E46, E113, and E117? As shown in Fig. 3D,E, a single or double mutation E46A and E113A can significantly rescue this short DNA phenotype by generation of a higher MW species of HBV specific DNA by Southern blot or PCR analysis. Due to some unknown poison effect of E117A, its overall rescue efficiency in viral DNA synthesis is greatly reduced. The rescue effect on RNA encapsidation is most evident, when double mutations E46A + E113A were used to rescue the short RNA phenotype of mutant ARD-I + II + III + IV by Northern blot and RT-PCR analyses (Fig. 3F,G). The rescued phenotypic effect of double mutations E46A + E113A can also be confirmed by Whole Genome PCR sequencing (Fig. 3H,I). Comparing to parental ARD-I + II + III + IV, charge rebalanced mutant E46A + E113A can generate higher MW DNA species. The significantly enhanced rescue effect of the double mutation E46A + E113A, relative to the single mutation E46A or E113A, strongly suggest that charge rebalance can be used to rescue charge-imbalanced mutants in a dose-dependent manner.

To test further the effect of other acidic residues on electrostatic interactions, we performed charge rebalance experiment using E40A and E180A mutations in the context of mutant ARD-III + IV (Fig. 3B and *upper panel*, 3 J). E180 is located near the carboxyl-terminus of HBc with no known structure^18^,^57^. In a dose dependent manner, the short DNA phenotype of mutant ARD-III + IV can be progressively rescued to generate longer and higher MW DNA species by single, double and triple rescue mutations. This E180A mutation exerted a highly potent rescue effect by Southern blot analysis (Fig. 3J, lane 2 vs. 6, lane 5 vs. 9, lane 8 vs. 11). Such a potent rescue effect on viral RNA encapsidation can be confirmed by Northern blot analysis (Fig. 3K, lane 1 vs. 5, lane 4 vs. 8, lane 7 vs. 10). In general, the rescue of full-length viral RNA encapsidation tended to be more efficient than the rescue of full-length RC DNA in charge rebalance experiments (lanes 8, 10, 11 of Fig. 3K vs. lanes 9, 11, 12 of Fig. 3J). As a control, we compared various single charge rebalance mutations in the wild type HBV context (Supplementary Figure S3E). We noted that only a minor effect on viral DNA synthesis of single mutants E40A, E46A and E117A, and no significant effect was detected in mutants E113A and E180A. Our results, based on assays for both viral RNA encapsidation and DNA synthesis, indicate that acidic residues of HBc from either N-terminus (E40, E46, and E113) or C-terminus (E180) can all contribute negative charge to the electrostatic interactions in the capsid interior in a dose dependent manner.

### Serine phosphorylation and de-phosphorylation play a role as a charge balance buffer

**Figure 4:**
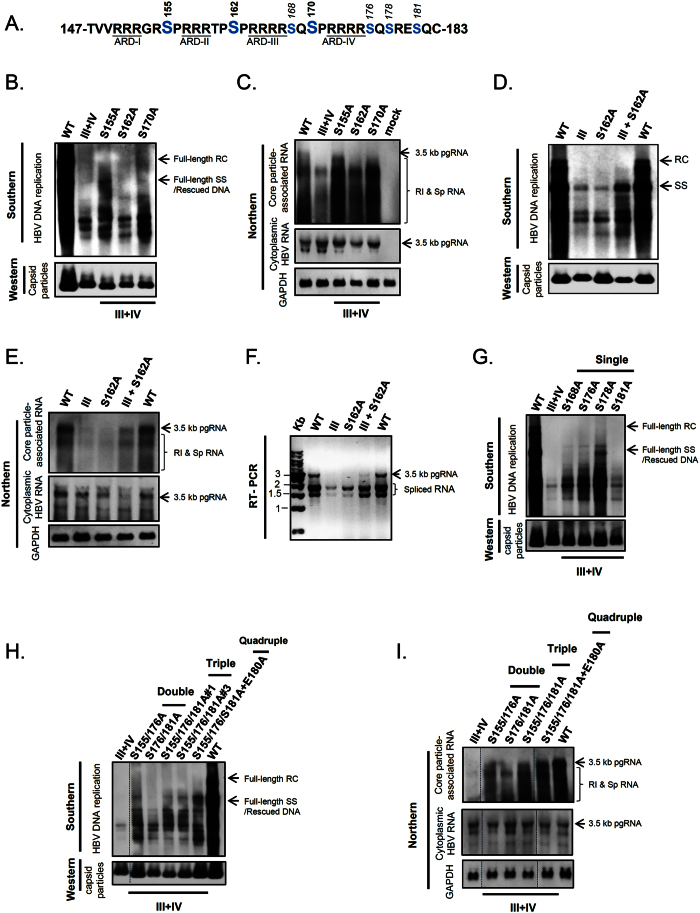
A charge rebalance approach via reducing serine phosphorylation at HBc ARD. **(A)** HBc ARD contains 7 potential serine phosphorylation sites (blue). **(B)** The replication defect of mutant ARD-III + IV can be partially rescued via a single S-to-A mutation at S155 and S170 by Southern blot analysis. **(C)** The short RNA phenotype of mutant ARD-III + IV can be significantly rescued via a single S-to-A mutation at S155, S162, S170 by Northern blot analysis. Mock: untransfected HuH-7 cells. Total cytoplasmic HBV RNA and GAPDH RNA were included as controls. **(D)** The replication defect of mutant ARD-III can be partially rescued by a charge rebalance mutation S162A by Southern blot analysis. Combining mutations S162A and ARD-III efficiently restored the full-length SS DNA, albeit no full-length RC DNA (see Discussion). **(E)** The defect in viral RNA packaging of mutant ARD-III can be efficiently rescued by a second mutation S162A by Northern blot analysis. Total cytoplasmic HBV RNA and GAPDH RNA were included as controls. **(F)** The Northern result in (**E**) can be confirmed by RT-PCR analysis. **(G)** The replication defect of mutant ARD-III + IV can be rescued, in the increasing order of efficiency, via single mutations S181A, S168A, S176A, and S178A at minor phosphorylation sites, by Southern blot analysis. **(H)** The defective DNA replication of HBc mutant ARD-III + IV can be rescued in a dose-dependent manner by combinations of multiple rebalance mutations S155A, S176A, S181A, and E180A. In triple-rescue mutants, clones #1 and #3 are two independent *E. coli* isolates. All the lanes shown here are from the same gel. **(I)** The RNA encapsidation defect of HBc mutant ARD-III + IV can be rescued in a dose-dependent manner via combinations of multiple rebalance mutations S155A, S176A, S181A, and E180A, by Northern blot analysis. HBV total cytoplasmic RNA and GAPDH were included as controls. All the lanes shown here are from the same gel.

We next asked if serine phosphorylation/de-phosphorylation at HBc could contribute to charge balance in the capsid interior. As mentioned in the Introduction, HBc has three major phosphorylation sites S155, S162 and S170^30^,^34^. Amino acids S162 and S170 had been shown to be important for HBV RNA encapsidation and DNA replication^37^,^38^,^41^,^58^ (Fig. 4A). To test the potential role of serine phosphorylation in balancing electrostatic interactions, we examined whether the phenotypic defect of arginine deficient mutants can be rescued by reducing negative charge at major phosphorylation sites.

We generated serine-to-alanine (S-to-A) substitutions which mimicked constitutive serine de-phosphorylation at major phosphorylation sites of mutant ARD-III + IV. Indeed, the short DNA phenotype of mutant ARD-III + IV can be rescued to produce longer DNA species by mutations S155A and S170A (Fig. 4B). Furthermore, the total amount of encapsidated RNA can be strongly increased in signal intensities by each individual mutation at all three major phosphorylation sites. Mutation S155A exhibited the most apparent increase in the size of encapsidated RNA (Fig. 4C). Our results revealed that the rescue effects from these three major phosphorylation sites are not identical. Although S155 itself has never been shown to play any essential role in RNA encapsidation^37^,^38^,^41^,^58^, our result here suggests that S155 could play a regulatory role in buffering charge imbalance in the capsid interior.

In addition to ARD-III + IV, we tested the rescue effect of mutation S162A in the context of mutant ARD-III (Fig. 4D–F). Individual mutations ARD-III or S162A have each been shown to abolish HBV DNA replication and RNA encapsidation^25^,^37^,^50^,^58^. Surprisingly, a double mutation of ARD-III and S162A efficiently restored the full-length single-strand (SS) DNA (Fig. 4D), and significantly increased the encapsidation efficiency of viral RNAs (Fig. 4E), including spliced RNAs (Fig. 4F). In a reciprocal manner, the full-length SS DNA of mutant S162A can be efficiently rescued by individual mutations at ARD-I, -II, -III, and -IV, respectively (Supplementary Figure S4). RNA encapsidation is known to be strongly dependent on phosphoserine-162^37^,^38^,^41^,^58^. Most strikingly, the pleiotropic defects, resulting from the de-phosphorylated serine 162, can be partially rescued by arginine deficiency at HBc ARD in a position-independent manner. Our results strongly suggest that major phosphorylated serines can interact with positive charge from HBc ARD in modulating electrostatic homeostasis.

In addition to these three major phosphorylation sites at S155, S162, and S170, there are known minor phosphorylation sites at S168, S176, and S178 (Fig. 4A)^30^,^31^. It remains unknown if S181 can be phosphorylated. We asked whether these minor phosphorylation sites could contribute to charge balance. In Fig. 4G, except for the moderate degree of rescue by S-to-A substitution at S181, other mutations S168A, S176A, and S178A partially rescued the severe defect in viral DNA replication of mutant ARD-III + IV, albeit no full-length RC form can be completely restored by any of these charge rebalance mutations. In fact, full-length RC form DNA has never been restored, even when we increased the “dose” of the charge rebalance effect by random combinations from multiple S-to-A and E-to-A mutations (Fig. 4H). In contrast to DNA rescue, encapsidated RNAs of mutant ARD-III + IV can be more efficiently rescued by combinations of multiple charge rebalance mutations (Fig. 4I). Taken together, our results indicate that serine phosphorylation at HBc ARD could contribute to charge balance in regulating HBV RNA encapsidation, DNA synthesis, and genome maturation.

### The puzzle of empty capsids in the *baculovirus* system

The charge balance hypothesis predicts that the encapsidated RNA is important for capsid stability by neutralizing the inter-subunit positive charge repulsion from the adjacent arginine residues^40^,^49^. However, it has been reported previously that HBV capsids produced from the *baculovirus* system contained no encapsidated RNA^47^. It is therefore puzzling how these empty capsids can maintain their capsid stability without any negative charge from the encapsidated RNA?

**Figure 5:**
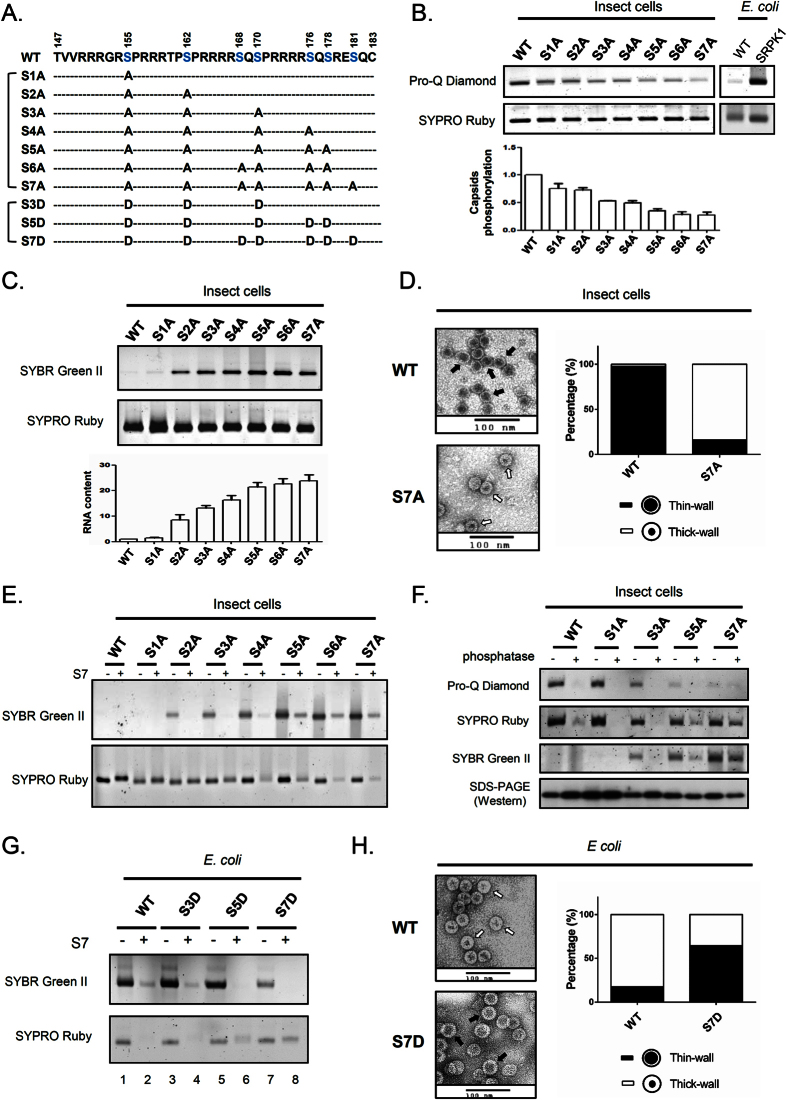
Generation of empty capsids in *baculovirus* and *E. coli* systems via hyper-phosphorylation of HBc. **(A)** Amino acid sequences of HBc ARD of wild type HBV and ten serine-deficient mutants. Blue residues indicate 7 potential serine phosphorylation sites. Alanine (**A**) and aspartic acid (**D**) substitutions mimic de-phosphorylation and phosphorylation. **(B)** *Upper panel:* The phosphorylation contents between WT and serine-deficient mutant capsids were compared by agarose gel electrophoresis. Pro-Q Diamond was used for phosphorylated protein staining^59^,^60^,^61^ and SYPRO Ruby was used for total protein staining^49^. The faint signal of WT from *E. coli* represents non-specific background noise. Capsid particles purified from *E. coli* co-expressing HBc and SRPK1 (SR protein-specific kinase 1) was included as a positive control for phosphoprotein. *Lower panel*: Quantitative comparisons of serine phosphorylation between WT and serine-deficient capsids by measuring the ratios between Pro-Q Diamond signals and SYPRO Ruby signals. **(C)** *Upper panel*: Reduction in serine phosphorylation of HBc capsids can convert WT empty capsids with no RNA into RNA containing capsids by SYBR Green II staining. *Lower panel*: Quantitative comparisons of the RNA/protein ratios among WT and serine-deficient mutant capsids. **(D)** Capsid particles of WT and serine-deficient mutants were stained with uranium acetate under electron microscopy. The numbers of “thin-wall” and “thick-wall” capsids were scored in a double-blind manner as reported previously^15^,^49^. WT capsids contain predominantly thin-wall morphology (*upper panel, black arrow*), while mutant S7A contain predominantly thick-wall capsids (*lower panel, white arrow*). **(E)** Capsid stability was compared between WT HBc and serine-deficient mutants by micrococcal nuclease S7 treatment. **(F)** Capsid stability was compared between WT HBc and serine-deficient mutants by phosphatase treatment. **(G)** *E. coli*-expressed capsids of WT HBc and phosphorylation-mimicking mutants were treated with micrococcal nuclease S7. **(H)** *E. coli*-expressed capsid particles of WT and mutant S7D were examined under EM and scored as described in (**D**). WT capsids contain predominantly thick-wall morphology (*upper panel,* white arrow), while S7D capsids contain predominantly thin-wall capsids (*lower panel,* black arrow).

To investigate on this puzzle, we tested the possibility whether serine phosphorylation of HBV core protein could substitute for the negative charge of RNA in maintaining the charge balance and capsid stability. We engineered a series of core mutants with increasing number of S-to-A substitutions into potential phosphorylation sites at ARD using the *baculovirus* core protein expression system (Fig. 5A). Pro-Q Diamond is a staining dye specific for phosphoprotein^59^,^60^,^61^. As expected, Pro-Q Diamond signals of HBc capsid particles gradually decreased from WT to mutant S7A (*upper panel*, Fig. 5B). These capsid particles of WT and serine-deficient mutants were further analyzed by staining with SYBR Green II for RNA and SYPRO Ruby for total protein (Fig. 5C). Consistent with the previous report^47^, WT HBc particles from insect cells were empty with no encapsidated RNA. However, to our surprise, mutant capsids S2A–S7A began to exhibit detectable amount of packaged RNA. The RNA contents for each mutant capsid, after normalization with their respective protein contents, appeared to be increasing from S2A to S5A, and reached plateau for S5A, S6A and S7A. The gradually increasing RNA contents appeared to be correlated with the increasing numbers of alanine substitution (de-phosphorylation) (*upper panel*, Fig. 5C). The results here support the conclusion that empty WT particles are hyper-phosphorylated. In addition, the inverse correlation between the degree of phosphorylation and the amount of encapsidated RNA supports the charge balance concept that phosphoserine can substitute for encapsidated RNA in electrostatic interactions.

As shown in Fig. 5D, under electron microscopy (EM) with negative staining, most of the WT particles with no encapsidated RNA displayed a “thin-wall” morphology, while mutant S7A with encapsidated RNA exhibited a predominantly “thick wall” morphology. These two types of morphology are reminiscent of our previous finding using *E. coli*-expressed capsid particles^15^. *E. coli*-expressed capsids can be disassembled by ribonuclease S7^49^. We tested here if this same phenomenon can be extended to *baculovirus*-expressed capsids. Indeed, when we treated WT and S-to-A mutant capsids with micrococcal nuclease S7, we observed capsid disassembly in mutants S3A to S7A (Fig. 5E). In contrast, no capsid disassembly was observed in WT, mutants S1A and S2A. Since WT, S1A and S2A capsids contain little amount of RNA, it is not surprising that they were resistant to nuclease digestion.

Encouraged by the nuclease digestion experiments, we asked if one can demonstrate biochemically the importance of phosphorylation in charge balance and capsid stability. We treated WT and mutant capsids with λ-phosphatase^25^ and monitored the digestion of phosphate group by Pro-Q Diamond (Fig. 5F). As expected, the Pro-Q Diamond staining intensities decreased progressively from WT to mutant S7A. Upon digestion with λ-phosphatase, capsid disassembly was induced in WT and most mutants on agarose gel by SYPRO Ruby staining. In contrast, mutant S7A appeared to be more resistant to phosphatase treatment. Capsid disassembly revealed by the SYPRO Ruby staining was confirmed by the RNA staining assay using SYBR Green II. As a control for sample loading, SDS-PAGE and Western blot analysis of HBc protein monomers were included (*lower panel*, Fig. 5F). As summarized in Supplementary Figure S5A, the puzzle of *baculovirus*-expressed empty capsids can be explained by the hyper-phosphorylation of HBV capsids, and this explanation can be verified experimentally by using a genetic approach of S-to-A mutants, and a biochemistry approach using λ-phosphatase and S7 nuclease digestions.

### Formation of empty capsids in *E. coli* via S-to-D mutations

Unlike the *baculovirus* system, an *E. coli* system produced mainly RNA-containing capsids^12^,^15^,^57^. Since the *E. coli* expression system is known to have no phosphorylation, we asked if multiple S-to-D substitutions at the ARD could mimic hyper-phosphorylation of HBc, and thus result in formation of empty particles. We expressed WT and various S-to-D HBc mutants in *E. coli* (Fig. 5A). Indeed, the total amount of encapsidated RNA was significantly decreased in S7D capsids by SYBR Green II staining (*upper panel*: lane 7, Fig. 5G). Upon digestion with S7 nuclease, capsid disassembly was induced in WT, mutants S3D and S5D by SYPRO Ruby staining (*lower panel*: lanes 2, 4, and 6, Fig. 5G). In contrast, mutant S7D capsids were far more resistant to S7 treatment, even when the nuclease digestion of encapsidated RNA was rather complete (lane 8, Fig. 5G). Under EM with negative staining, most of WT capsids displayed “thick-wall” morphology (RNA-containing capsids), while S7D capsids displayed “thin-wall” morphology (empty capsid) (Fig. 5H). Taken together, we reproduced successfully the thin-walled empty capsids in *E. coli* by introducing multiple phosphorylation-mimicking mutations (S7D) into the ARD of HBc (Supplementary Figure S5B).

### Serine phosphorylation and HBV RNA packaging in the context without active viral replication in human hepatocytes

**Figure 6:**
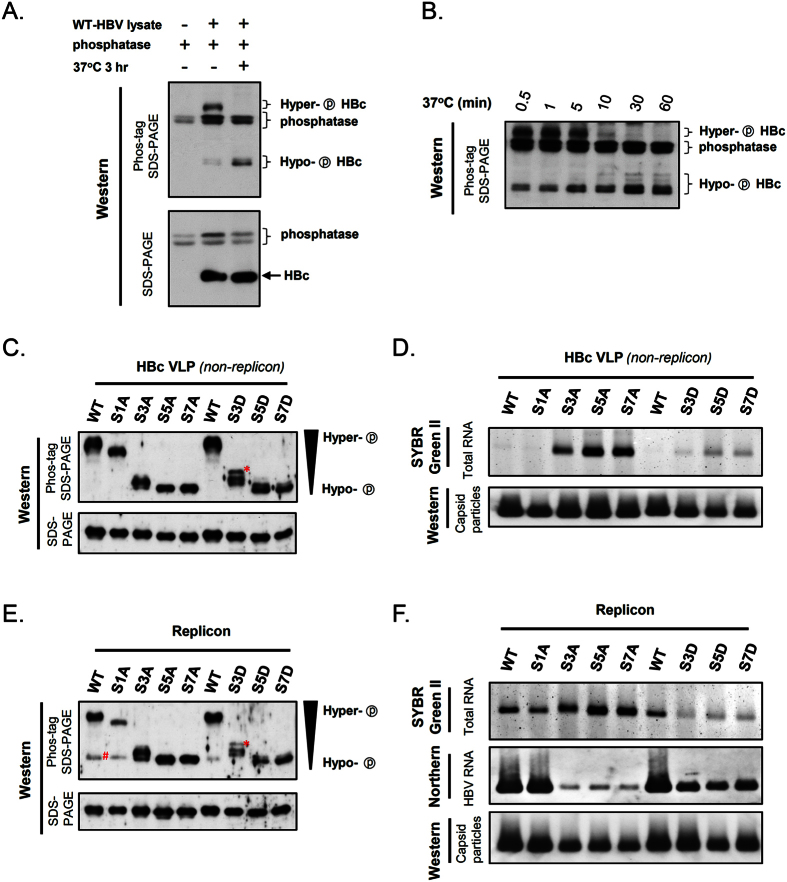
Evaluation of the effect of serine phosphorylation at HBc ARD on capsid assembly and RNA packaging in replicon and non-replicon contexts. **(A)** *Upper panel*: Upon phosphatase treatment, hyper-phosphorylated HBc of a WT-HBV replicon downshifted to a faster-migrating position on the Phos-tag SDS-PAGE. Mobility of phosphoproteins on this gel assay is determined by the degree of phosphorylation^62^,^63^,^64^. *Lower panel*: In contrast, the phosphorylation status of HBc cannot be resolved by the regular SDS-PAGE. **(B)** In a time course experiment with phosphatase digestion (0.5 - 60 min), a gradual downshift pattern of WT-HBc protein was monitored on the Phos-tag gel. **(C)** *Upper panel*: In the non-replicon context in HuH-7 cells, the phosphorylation status of HBc ARD from WT and serine phosphorylation mutants was assessed by using the Phos-tag gel assay. *Lower panel*: As an internal control, the same samples were analyzed by regular SDS-PAGE and Western blot. *This band is reproducibly present in S3D, but not in S3A (see Discussion). **(D)** *Upper panel*: RNA contents of HBc VLPs (non-replicon) were analyzed by staining with SYBR Green II. *Lower panel*: Assembled capsids were included as an internal control and visualized by Western blot analysis. **(E)** In the HBV replicon context in HuH-7 cells, the phosphorylation status of HBc ARD from WT and serine phosphorylation mutants was analyzed by Phos-tag SDS-PAGE. As an internal control, the same samples were analyzed by regular SDS-PAGE and Western blot. *This band is reproducibly present in S3D, but not in S3A (see Discussion). ^#^This band is present only in the replicon system (compare Fig. 6C,E). (**F**) In the HBV replicon context, HBV capsids were measured by native gel and stained with SYBR Green II (*Upper panel)*, and assayed by Northern blot by using a 3.2 kb HBV-specific probe (*Middle panel*) and Western blot by using anti-HBc Ab (*Lower panel)*.

Finally, in addition to insect cells and *E. coli,* we asked if the relationship between serine phosphorylation and charge balance can be examined in human hepatocytes, with or without active viral replication. So far, we have relied on a genetic approach to study the effect of serine phosphorylation by phosphorylation- or de-phosphorylation-mimicking mutations (Figs 4 and 5). To directly monitor the phosphorylation status of HBc ARD, we turned to a quantitative Phos-tag gel assay for a more direct assessment of protein phosphorylation (Fig. 6A–C). In this assay, hyper-phosphorylated proteins would be retarded in mobility due to the binding of Phos-tag in the gel to the phosphate groups of phosphorylated proteins^62^,^63^,^64^. As shown in Fig. 6A, *upper panel*, a WT-HBV replicon was transfected into HuH-7 cells. Hyper-phosphorylated HBc proteins in the cell lysates were treated with phosphatase at 37 °C for 3 hr, resulting in a dramatic downshift banding pattern of hypo-phosphorylated HBc. In contrast, the conventional SDS-PAGE without Phos-tag was unable to resolve phosphorylated from de-phosphorylated proteins (*lower panel*, Fig. 6A). In a time course experiment with phosphatase treatment, gradual downshift of HBc was also observed on the Phos-tag gel assay (Fig. 6B). To validate further this Phos-tag gel assay, we compared the phosphorylation status of HBc VLP particles among WT and serine phosphorylation mutants (Fig. 6C). As expected, WT and mutant S1A capsids were hyper-phosphorylated, while mutants S3A, S5A, and S7A banded at the hypo-phosphorylated positions. We noted that HBc of mutants S3A was more phosphorylated than those of mutants S5A and S7A. Similarly, mutant S3D was more phosphorylated than those of S5D and S7D (S-to-D mutations are phosphorylation-mimicking, without a *bona fide* phosphate group).

Next, we compared the encapsidated RNA contents among WT and mutants by SYBR Green II staining (Fig. 6D). Similar to the *baculovirus* system, WT and mutant S1A capsids with hyper-phosphorylated HBc were empty with no detectable RNA, while mutants S3A, S5A and S7A contained increasing amounts of packaged RNA. Again, the amount of encapsidated RNA correlated with the degree of ARD de-phosphorylation. In contrast to S-to-A mutants, S-to-D mutant capsids contained only trace amount of encapsidated RNA due to the excessive negative charge in the capsid interior (Fig. 6D). In summary, our results observed an inverse correlation between the degree of HBc ARD phosphorylation and the amount of encapsidated RNA in mammalian hepatocytes *in vivo*. Our studies here support the notion that empty particles without RNA are hyper-phosphorylated.

### Serine phosphorylation and HBV RNA packaging in the context with active viral replication in human hepatocytes

We also examined the relationship between serine phosphorylation and RNA encapsidation in the replicon context of viral replication (Fig. 6E,F). We compared the HBc phosphorylation status among WT and serine phosphorylation mutants by the Phos-tag gel analysis. The overall Phos-tag patterns are highly similar between the non-replicon (Fig. 6C) and replicon systems (Fig. 6E).

By comparing Fig. 6C,E closely, we noted the reproducible presence of a hypo-phosphorylated banding present only in the replicon system of WT and S1A (marked as # in Fig. 6E). Previously, it has been reported that serine phosphorylation at S162 or S170 is important for pgRNA encapsidation, while de-phosphorylation at these major sites may be important for viral DNA replication^37^,^38^,^39^,^41^. Therefore, this band # unique to the replicon system of WT and mutant S1A, could reflect a highly or near-completely de-phosphorylated HBc species required for viral replication.

**Figure 7:**
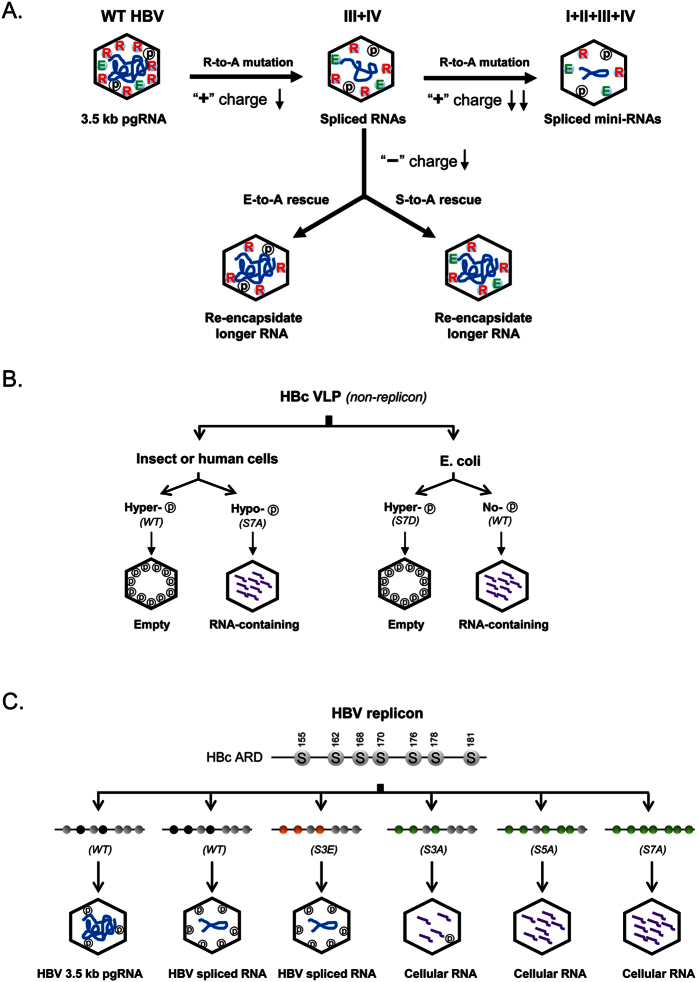
A cartoon summary of three major approaches used here in testing the HBV charge balance hypothesis. **(A)** The short RNA phenotype in arginine-deficient HBc mutants (e.g., ARD-III + IV) can be rescued successfully by using a charge rebalance approach (Fig. 3A). Both E-to-A and S-to-A mutations can restore the efficient packaging of longer-sized viral RNA, suggesting that serine phosphorylation at the ARD could contribute negative charge to electrostatic homeostasis required in the capsid interior (Figs 3 and 4, and Supplementary Figure S3). Similarly, mutant ARD-I + II + III + IV contains the most severe arginine deficiency and encapsidates predominantly spliced mini-RNAs of the shortest size (Figs 1 and 2). (**B**) Hyper-phosphorylation of HBc is responsible for the biogenesis and maintenance of empty capsids (without encapsidated RNA) in both insect and human cell systems (Figs 5 and 6). Empty capsids can also be expressed in *E. coli* by engineering a mutant S7D bearing 7 phosphorylation-mimicking aspartic acids at HBc ARD (Fig. 5). (**C**) WT HBV with double phosphorylations at S162 and S170 of HBc is believed to preferentially encapsidated 3.5 kb pgRNA^37^,^38^,^41^. In contrast, mutant S3E, containing three S-to-E mutations at the major phosphorylation sites (S155E, S162E and S170E) preferentially encapsidated spliced viral RNA^39^. Mutant S3A contains three de-phosphorylation mimicking mutations at three major phosphorylation sites (S155A, S162A, and S170A), and is known to be deficient in viral RNA encapsidation^37^,^38^,^41^. Interestingly, in the replicon context in HuH-7 cells, mutants S3A, S5A, and S7A can package increasing amounts of cellular RNA (Fig. 6F). Furthermore, we predict here that when all three major sites are phosphorylated, the 2.2 kb spliced RNA (1959 nt in Fig. 2E) will be more fitting for encapsidation than the non-spliced 3.5 kb pgRNA. Grey: wild type serine; black: phosphorylated serine; orange: S-to-E mutations; green: S-to-A mutations.

Next, we compared the RNA encapsidations among WT and phosphorylation mutants by SYBR Green II staining and Northern blot analysis. WT and S1A capsids contained abundant amount of encapsidated HBV RNA (*middle panel*, Fig. 6F). In contrast, mutants S3A, S5A and S7A packaged little amount of virus-specific RNA. This result alone appears to be opposite to the prediction from the charge balance hypothesis. However, when the total amount of encapsidated RNAs were measured by SYBR Green II (*upper panel*, Fig. 6F), surprisingly, the results are entirely consistent with the prediction from the charge balance hypothesis. We noted here an interesting inverse correlation between encapsidated *viral* RNA and encapsidated *total* RNA. While these S-to-A mutants encapsidated reduced amounts of HBV-specific RNAs (*middle panel*, Fig. 6F), they simultaneously packaged increased amounts of total RNA (*upper panel*, Fig. 6F). Based on the concept of charge balance hypothesis, it is tempting to speculate that more non-viral RNA could be encapsidated in compensation for a reduced level of encapsidated viral RNA in HBV RNA encapsidation, capsid assembly and stability (see Fig. 7C for Discussion). Indeed, such an inverse correlation between the negative charge content and RNA encapsidation was also observed in mutants S3D, S5D, and S7D (*upper panel*, Fig. 6F). Consistent with previous reports^37^,^38^,^39^,^58^, phosphorylation-mimicking S-to-D mutations at major phosphorylation sites allowed encapsidation of HBV-specific RNA in the replicon system, albeit the signals were reduced relative to those in WT and S1A mutant (see Discussion in Fig. 7C). In summary, balanced electrostatic interactions appeared to play a critical role in HBV capsid biology using both non-replicon and replicon systems in human hepatocytes.

## Discussion

As summarized in Fig. 7A, we invented a charge rebalance approach (Fig. 3A), and provided here experimental evidence for the charge balance hypothesis. Arginine-deficient HBV mutants in the full-length HBc context encapsidated shorter and spliced viral RNAs (Figs 1 and 2). This phenotype can be rescued to package longer or even normal-sized viral RNAs, by either reducing serine phosphorylation at the ARD or reducing negatively charged acidic residues within or outside ARD. This hypothesis remains highly robust, when tested via a charge rebalance approach in various HBc contexts of mutants ARD-III, ARD-IV, ARD-III + IV, and ARD-I + II + III + IV (Figs 3 and 4, and Supplementary Figure S3). Taken together, our results reinforced the concept that the *relative* amount of positive versus negative charge in electrostatic interactions, is more important than the *absolute* amount of positive or negative charge in HBV capsid stability, assembly, RNA encapsidation, and DNA synthesis. Furthermore, the diverse source of negative charge involved in electrostatic interactions could range from RNA, acidic amino acids, phosphoserines, oligonucleotides, poly-glutamic acid, sulfated polysaccharide, and even non-biopolymer polyacrylic acid^49^. Conversely, lysine (K) and arginine (R) are often substitutable positive charges for maintaining electrostatic balance in RNA encapsidation and DNA synthesis^25^,^40^ (Supplementary Figure S6). In brief, both polycations and polyanions can participate in electrostatic interactions important for maintaining capsid stability and assembly, irrespective of the exact chemical structure of the encapsidated materials.

Superficially, the existence of empty capsid or virion particles without any encapsidated RNA^42^,^43^,^44^,^45^,^46^, appears to present a challenge to the charge balance hypothesis^40^,^48^,^49^. We speculated that it is serine hyper-phosphorylation at the ARD that allows empty capsids to maintain balanced electrostatic interactions. Indeed, upon *in vitro* treatment with exogenous phosphatase, hyper-phosphorylated WT capsids from the *baculovirus* system collapsed into unassembled subunits (Fig. 5F). In contrast, mutant capsids S7A, encapsidated with abundant amount of RNA, were highly resistant to phosphatase-induced capsid disassembly. The exclusion of RNA encapsidation by HBc hyper-phosphorylation was further verified experimentally by engineering a phosphorylation-mimicking S7D HBc mutant in the *E. coli* system (Fig. 5G,H). Unlike the WT HBc expressed in *E. coli*, mutant S7D produced predominantly empty capsids in *E. coli,* reminiscent of those empty capsids produced in human hepatocytes HuH-7 (Fig. 6D). Empty capsids were implicated in a cell-free *in vitro* translation system and were observed in non-hepatocytes HEK293^65^. Again, from Fig. 7A,B, the sources of negative charge for balanced electrostatic interactions can include encapsidated nucleic acids, phosphoserines, and acidic residues. These three independent sources of negative charge are functionally substitutable for each other in the context of the charge balance hypothesis.

In Fig. 6F, in the replicon context, we observed an inverse correlation between viral RNA and total (including nonviral) RNAs in intracellular HBV capsids in human hepatocytes. HBV pgRNA encapsidation is known to be mediated by polymerase recognition of the packaging signal **ε** of pgRNA^66^,^67^,^68^,^69^. Moreover, double phosphorylations at both S162 and S170 of HBc are essential to efficient encapsidation of full-length pgRNA (Fig. 7C)^37^,^38^,^39^,^41^. Taken together from our RT-PCR data in WT HBV (Fig. 2A,B,E) and the reported studies on the three major phosphorylation sites^39^, we speculate here that a triple (n = 3) or multiple (n > 3) phosphorylation status of HBc could favor the encapsidation of spliced viral RNAs. Interestingly, when the three major phosphorylation sites are ablated in mutants S3A, S5A, and S7A, as expected, encapsidation of viral RNA is severely reduced^37^,^38^,^41^. However, coincidentally, we observed increased encapsidation of cellular RNA in a seemingly compensatory manner (Figs 6F and 7C). Again, this compensatory-like phenomenon lends another strong support for the charge balance hypothesis in the replicon system in human hepatocytes.

It is worth pointing out here that many spliced RNAs also contain the same packaging signal **ε** and can be recognized by the same polymerase as the non-spliced pgRNA. However, selectivity of RNA encapsidation is not solely or entirely dependent on the ε signal and polymerase, it is equally important to achieve balanced electrostatic interactions between the negative charge from encapsidated RNAs and HBc serine phosphorylation, and the positive charge from HBc ARD. For example, the size of encapsidated RNAs appeared to be determined by the arginine content of HBc ARD (Fig. 2). In summary, in addition to polymerase and ε, charge balance appeared to be a fundamental rule in selecting the optimum size and amount of encapsidatd viral nucleic acids. In addition to viral RNA and DNA, encapsidation of cellular RNAs could be another measure to fine tune the balanced electrostatic interactions in capsid assembly and stability in both replicon and non-replicon systems in hepatocytes.

It is worth discussing here the potential hierarchy in the position effects of HBc charged residues. We noted that not every positive or negative charged amino acids of HBc can make equal contributions to electrostatic interactions or biological consequences. For example, unequal effects from different acidic residues at different HBc positions were observed. In the charge-rebalance approach for mutant ARD-III + IV, the rescue effect of mutation E180A is the most potent than those at other acidic positions (Fig. 3J,K). A tentative hierarchy of the rescue effect on viral replication of mutant ARD-III + IV is E180A ≫ E113A > E40A > E46A. In our studies on serine phosphorylation, charge rebalance mutation S155A is the most efficient in restoring the size and the amount of encapsidated viral RNAs of mutant ARD-III + IV (S155A > S170A > S162A) (Fig. 4C). In the case of minor phosphorylation sites, the rescue efficiency goes in this order: S178A > S176A > S168A ≫ S181A (Fig. 4G). Finally, in our comparative studies on the arginine-rich subdomains (Fig. 1 and Supplementary Figure S1B), the importance in RNA encapsidation and DNA synthesis appears to follow this order: ARD-III > ARD-IV > ARD-II ≫ ARD-I (Fig. 1D)^25^,^50^, albeit each single ARD mutant contains exactly the same degree of arginine deficiency. Similarly, mutant ARD-III + IV displayed the strongest phenotypic defect relative to other double ARD mutants (Fig. 1). At present, no structure of HBc ARD by cryoEM or X-ray crystallography is available^18^,^70^,^71^. Nor do we know the folded structure of the encapsidated viral RNA in complexation with polymerase or core protein. In a thermodynamic model for genome packaging in hepatitis B virus, approximately 10% of the ARD residues are free from complex association with RNA^72^. A better understanding on the variable position effects of the various charged residues would have to depend on more detailed structural information about the ribonucleoprotein (RNP) complex of the encapsidated RNA, HBc ARD, and polymerase.

In our earlier charge rebalance experiment, we used a single acidic residue at the N-terminus of the truncated HBc^48^. Here, for the first time in a dose-dependent manner, we demonstrated that serine phosphorylation and E180A at the C-terminus of full-length HBc, can also contribute to electrostatic homeostasis. Combinations of multiple charge rebalance mutations are more effective than single charge rebalance mutation in the phenotypic rescue. Previously, we observed that mutation E117A can successfully rescue a replication-defective and C-terminus truncated HBc mutant 173GG^48^. In our current study using the full-length HBc, to our surprise, we found that mutation E117A is always highly deleterious to viral RNA encapsidation and DNA synthesis in various arginine-deficiency contexts, including ARD-III, ARD-IV, and ARD-I + II + III + IV, but not in WT context (Fig. 3 and Supplementary Figure S3). We speculate here that the glutamic acid at E117 might have important dynamic electrostatic interactions with the arginine residues in ARD-III and ARD-IV in HBV life cycle. Taken together, it is important to use full-length HBc context for HBV virological studies.

Wild type HBV and arginine-deficient mutants encapsidated many spliced viral RNAs detectable by RT-PCR, however, whose DNA counterparts cannot be found by PCR (Fig. 2 and Supplementary Figure S2). Conversely, some DNA species can be easily detected by PCR, yet, not easily detectable by RT-PCR.

The former case could result from at least two reasons: (1) Important sequence elements required for viral DNA synthesis^73^,^74^, may have already been deleted by RNA splicing in viral RNA biogenesis. (2) Because HBV replicates via reverse transcription of its pgRNA template, viral RNA encapsidation is an event upstream to viral DNA synthesis in HBV life cycle. In the charge rebalance experiments (Figs 3 and 4, and Supplementary Figure S3), the rescue of near-full-length RNA encapsidation was always more efficient than the rescue of near-full-length RC DNA synthesis. It is conceivable that DNA synthesis requires many additional steps following RNA encapsidation, such as primer translocation, template switch and nucleic acid chaperone activity^25^. Considering the pleiotropic defects in ARD mutants, it is not surprising that RNA encapsidation tends to be easier to be restored than RC DNA synthesis in charge rebalance experiments. Despite the fact that the molecular mechanisms of RNA encapsidation and DNA synthesis are not the same, they both comply to the charge balance rule. For example, HBc de-phosphorylation during genome maturation^32^,^36^ could be for the purpose of maintaining electrostatic homeostasis.

In the latter case, we detected some abundant DNA species by PCR assay, yet not as abundant by RT-PCR assay. For example, the full-length 3.2 kb DNA can be identified as a highly abundant species by PCR cloning and sequencing, yet, the full-length 3.2 kb cDNA by RT-PCR can be hardly detected (Fig. 2E). We attributed this difference as due to the inefficiency of reverse transcriptase *in vitro* due to its notorious non-processive nature.

It is known that HBc ARD consists of major and minor phosphorylation sites^30^,^31^,^34^ (Fig. 4A). It remains an open issue whether these multiple phosphorylation events in HBc ARD, could occur in a random or stochastic manner, or an ordered or programmed manner in hepatocytes. Is there any phosphorylation cascade at HBc ARD? While this is a complicated issue, it is clear that phosphorylation at any of these three major phosphorylation sites (S155, S162, and S170) is not a prerequisite to the phosphorylation at other minor phosphorylation sites at ARD. In both replicon and non-replicon systems (Fig. 6C,E), we observed further increased phosphorylation of mutant S3A, relative to mutants S5A and S7A, indicating that phosphorylation at minor sites (S176 and S178) could occur even when major sites of mutant S3A are already genetically blocked. We also noted a further phosphorylated banding present only in mutant S3D, but not in S3A (marked with * in Fig. 6C,E). This result can be interpreted as phosphorylation-mimicking at major sites could predispose the phosphorylation at minor sites. This interpretation is consistent with the prediction in a recent report that phosphorylation at major sites may facilitate the phosphorylation at minor sites^65^. In summary, our Phos-tag assay results strongly support the notion that phosphorylations at major sites are not necessary, yet it could be sufficient, for the phosphorylation at minor sites.

In our charge balance hypothesis, we highlights the importance of the stoichiometry between positive and negative charges in the capsid interior in HBV life cycle^40^,^48^,^49^. By theoretical calculation, it was proposed that a fixed and conserved universal ratio (N/Q = 1.61) exists between the viral genome size (N) and the net charge of the capsid protein (Q)^75^. This fixed ratio hypothesis appeared to be based on three assumptions: (1) Both capsid protein and encapsidated RNA or DNA are linear (non-folded) polyelectrolytes; (2) Capsid proteins are not post-translationally modified; (3) Viruses exist as a single homogeneous population in nature. In our case of HBc ARD, there is no known structure by cryoEM or X-ray diffraction^18^,^70^,^71^. In addition, HBc ARD can be highly phosphorylated and HBV exists as a highly heterogeneous population (e.g., T = 3 vs. T = 4 icosahedral particles). Recently, by theoretical calculation, Kim and Wu found that the amount of encapsidated RNA is not linearly correlated with the net charge of the cytoplasmic tail of HBV capsid protein^72^,^76^. Similar findings in favor of a non-fixed ratio (N/Q) were reported in other viral systems^77^,^78^,^79^,^80^.

In conclusion, in addition to HBV, we anticipate that a similar mechanism of balanced electrostatic interactions in the capsid interior could serve as a guiding principle for other non-HBV viruses in nature.

## Methods

### Plasmids and Cell lines

HBc ARD mutants were engineered from the parental replicon plasmid pCHT-9/3091^81^, which contains a 1.1-mer HBV *ayw* genome under a CMV promoter. Site-directed mutagenesis was conducted using the QuickChange XL Site-Directed Mutagenesis Kit (Agilent Technologies, Santa Clara, CA, USA). All mutants were confirmed by DNA sequencing. HuH-7 hepatoma cell line was obtained from Dr. M. Lai at Academia Sinica, Taiwan^82^.

### Assays for HBV core-associated DNA and RNA

Experimental procedures for the extractions of encapsidated viral RNA and DNA, as well as Southern and Northern blot analyses, are as described previously^40^,^48^,^83^. Plasmids of wild-type HBV or HBc mutants in HBV-replicon (pCHT-9/3091) were co-transfected with plasmid pMT-pol (an HBV polymerase expression vector)^84^ into human HuH-7 cells using PolyJet transfection reagent (SignaGen Laboratories, Rockville, MD, USA). Capsid particles were prepared using the PEG precipitation method^85^,^86^,^87^.

### Native agarose gel and Western blot of core particles

Experimental procedures for native agarose gel electrophoresis and immunoblot are as described elsewhere^48^. Rabbit anti-HBc polyclonal antibody (DAKO, Glostrup, Denmark) was used in 1:2000 dilutions, while goat anti-rabbit-IgG-HRP antibody (GeneTex Inc, Taiwan) was used in 1:4000.

### RT-PCR-sequencing

For RT-PCR, 55 μM of oligo (dT)_18_ and 5 μg of core-associated RNAs were used for reverse transcription reaction by SuperScript III RT kit (Invitrogen, Thermo Fisher Scientific, Waltham, MA, USA). To amplify HBV *ayw* cDNA, the PCR reaction was mixed with forward primer 1818 S (5′-GCAACTTTTTCACCTCTGCC-3′), reverse primer 1824AS (5′-AAGTTGCATGGTGCTGGTGCGCA-3′) and 2 unit of Platinum Taq DNA Polymerase High Fidelity (Invitrogen, Thermo Fisher Scientific, Waltham, MA, USA). PCR amplification was performed for 35 cycles. Each cycle consists of denaturation at 94 °C for 30 sec, annealing at 55 °C for 30 sec, and elongation at 72 °C for 6 min. Finally, cDNAs were subcloned into the TOPO TA cloning vector (Invitrogen, Thermo Fisher Scientific, Waltham, MA, USA) and confirmed by DNA sequencing. The DNA sequences were analyzed by Spidey (NCBI, http://www.ncbi.nlm.nih.gov/spidey/). Data and statistical analysis were performed with GraphPad Prism Software version 6.0 (GraphPad software, San Diego, California, USA).

### PCR of Whole Genome DNA and sequencing

Preparations of intracellular and extracellular viral DNAs were as described previously^28^,^83^. HBV core-associated DNAs were amplified by PCR with HBV Whole Genome primers^10^: forward primer (nt 1821 to 1841), CCGGAAAGCTTGAGCTCTTCTTTTTCACCTCTGCCTAATCA and reverse primer (nt 1823 to 1806), CCGGAAAGCTTGAGCTCTTCAAAAAGTTGACTGGTGCTGG and Platinum Taq DNA Polymerase High Fidelity (Invitrogen, Thermo Fisher Scientific, Waltham, MA, USA). The PCR reactions were performed in two steps. In the first 10 cycles, denaturation was at 95 °C for 40 sec, annealing at 60 °C for 1.5 min, and elongation at 72 °C for 3 min. In the next 15 cycles, elongation was conducted by increasing 12 sec in each cycle (T300, Thermocycler, Bio-Rad, Hercules, CA, USA). Finally, the PCR-amplified DNAs were subcloned and analyzed as described above for RT-PCR amplified DNA.

### *Baculovirus*-expressed HBc capsids in insect cells

The full-length WT HBc and various HBc mutants S1A to S7A were cloned into pFastBac1 expression vector at *EcoRI* and *NotI* restriction sites (Thermo Fisher Scientific, Waltham, MA, USA). All mutants were confirmed by DNA sequencing. *Spodoptera frugiperda* 9 (Sf9) cells were purchased from BCRC (Bioresource Collection and Research Center, Taiwan; ATCC number: CRL-1711)^88^. and were grown at 28 °C in Protein-Free Insect Cell Culture Medium ESF 921 (Expression Systems, Davis, CA, USA). The recombinant *baculoviruses* containing various HBc mutants were generated with the Bac-to-Bac *baculovirus* expression system (Thermo Fisher Scientific, Waltham, MA, USA), and amplified in Sf9 insect cells as described^89^. The supernatants were harvested 3 days post-infection. HBc capsid-containing media were pelleted through a 20% sucrose cushion in 1X Tris-buffered saline (TBS) (0.1 M NaCl, 2 mM KCl, 25 mM Tris pH 7.4) at 26,000 rpm for 18 hr (SW28, Beckman). For further purification, HBc capsids were loaded on a continous 40–70% sucrose gradient at 26,000 rpm for 18 hr (SW28, Beckman). Fractions were collected and analyzed by Western blot with anti-HBc antibody (DAKO, Glostrup, Denmark). HBc-containing fractions were then pooled together, and concentraed through a 20% sucrose chusion at 26,000 rpm for 16–18 hr (SW28, Beckman). The HBc capsid pelletes were resolved in 1X TBS.

### *E. coli* - expressed HBc capsids

HBc mutants S3D, S5D, and S7D were generated with a QuickChange XL Site-Directed Mutagenesis Kit (Agilent Technologies, Santa Clara, CA, USA), using the full-length wild-type (*ayw*) plasmid pET-Blue-1-HBc183 as a template. All mutants were confirmed by DNA sequencing. WT and various HBc mutants were expressed in *E.coli* and purified by a 40–70% sucrose gradient^49^. The HBc capsid-containing fractions were concentrated using a 20% sucrose cushion, and the purified capsids were resolved in 1X TBS.

### Preparation of mutant capsids in human hepatoma HuH-7 cells

For the HBc VLP system (non-replicon), mutants S1A, S3A, S5A, S7A, S3D, S5D and S7D were generated with a QuickChange XL Site-Directed Mutagenesis Kit (Agilent Technologies, Santa Clara, CA, USA), using the plasmid pFastBac-CMV-HBc WT as a template. DNA plasmids of WT and various HBc mutants were transfected into HuH-7 cells. Three days post-transfection, intracellular HBc capsid particles were purified using a 25% sucrose cushion and resolved in 1X TBS. For the HBV replicon system used in Fig. 6, DNA plasmids of pFastBac-CMV-HBc WT and its derivative serine phosphorylation mutants were co-transfected with pCHT-9/3091-1903 (defect in HBc initiation codon) into HuH-7 cells. Five days post-transfection, intracellular HBc capsid particles were purified using a 25% sucrose cushion and resolved in 1X TBS.

### Capsid disassembly by micrococcal nuclease or λ-phosphatase treatments

The protocol of capsid disassembly by micrococcal nuclease treatment was as detailed elsewhere^49^. The purified capsids in TBS was diluted 5–20 fold in distilled H_2_O before incubation overnight at 37 °C with micrococcal nuclease (New England BioLabs, Ipswich, MA, USA) in the presence of 8 mM CaCl_2_ and 5 mM dithiothreitol (DTT). De-phosphorylation was conducted by incubation with λ-phosphatase (END Millipore, Darmstadt, Germany) at 37 °C for 3.5 hr in the presence of 50 mM HEPES, PH 7.5, 0.1 mM EDTA, 10 mM MnCl_2_ and 5 mM DTT. Complete digestion by micrococcal nuclease or λ-phosphatase was monitored on 1% native agarose gel, using SYBR Green II staining for encapsidated nucleic acids, SYPRO Ruby for capsid proteins^49^ and Pro-Q Diamond (Invitrogen, Thermo Fisher Scientific, Waltham, MA, USA) for protein phosphorylation^59^,^60^,^61^. The images were scanned with a Typhoon 9410 Mode Imager (Amersham BioSciences Corp., Piscataway, NJ, USA) and the banding intensity was quantified by Image J software (National Institutes of Health).

### Phos-tag SDS-PAGE

HBV capsids were mixed with 5X SDS-loading dye (5% β-Mercaptoethanol, 0.02% Bromophenol blue, 30% Glycerol, 10% SDS, 250 mM Tris-HCl, PH 6.8) and boiled for 10 min before loading onto polyacrylamide-bound Mn^2+^-Phos-tag SDS-PAGE (Wako Pure Chemical Industries, Richmond, VA, USA)^62^,^63^,^64^. After electrophoresis, the gel was soaked for 10 min in protein transfer buffer (25 mM Tris, 192 mM glycine, 10% methanol) containing 1 mM EDTA, followed by soaking for another 10 min in the same buffer without EDTA. Western blot analysis was performed by using rabbit anti-HBc polyclonal antibody (DAKO, Glostrup, Denmark).

## Additional Information

**How to cite this article**: Su, P.-Y. *et al*. HBV maintains electrostatic homeostasis by modulating negative charges from phosphoserine and encapsidated nucleic acids. *Sci. Rep.* **6**, 38959; doi: 10.1038/srep38959 (2016).

**Publisher’s note:** Springer Nature remains neutral with regard to jurisdictional claims in published maps and institutional affiliations.

## Supplementary Material

Supplementary Information
