# Supplementary material for: HBV maintains electrostatic homeostasis by modulating negative charges from phosphoserine and encapsidated nucleic acids
Source: Sci Rep. 2016 Dec 13;6:38959. doi: 10.1038/srep38959 (PMC5154190; doi:10.1038/srep38959)
Supplement: Supplementary Information [file srep38959-s1.pdf]

Supplementary Information

HBV maintains electrostatic homeostasis by modulating negative charges from phosphoserine and encapsidated nucleic acids

Authors: Pei-Yi Su[1,2,3], Ching-Jen Yang[2], Tien-Hua Chu[2,4], Chih-Hsu Chang[2,5], Chiayn Chiang[2], Fan-Mei Tang[2], Chih-Yin Lee[2], Chiaho Shih[2*]

[1]Taiwan International Graduate Program in Molecular Medicine, National Yang-Ming University and Academia Sinica, Taipei, Taiwan;

[2]Institute of Biomedical Sciences, Academia Sinica;

[3]Institute of Biochemistry and Molecular Biology, National Yang-Ming University, Taipei, Taiwan;

[4]National Defense Medical Center, Taipei, Taiwan

[5]Graduate Institute of Microbiology, College of Medicine, National Taiwan University, Taipei, Taiwan

* Corresponding author

E-mail: cshih@ibms.sinica.edu.tw

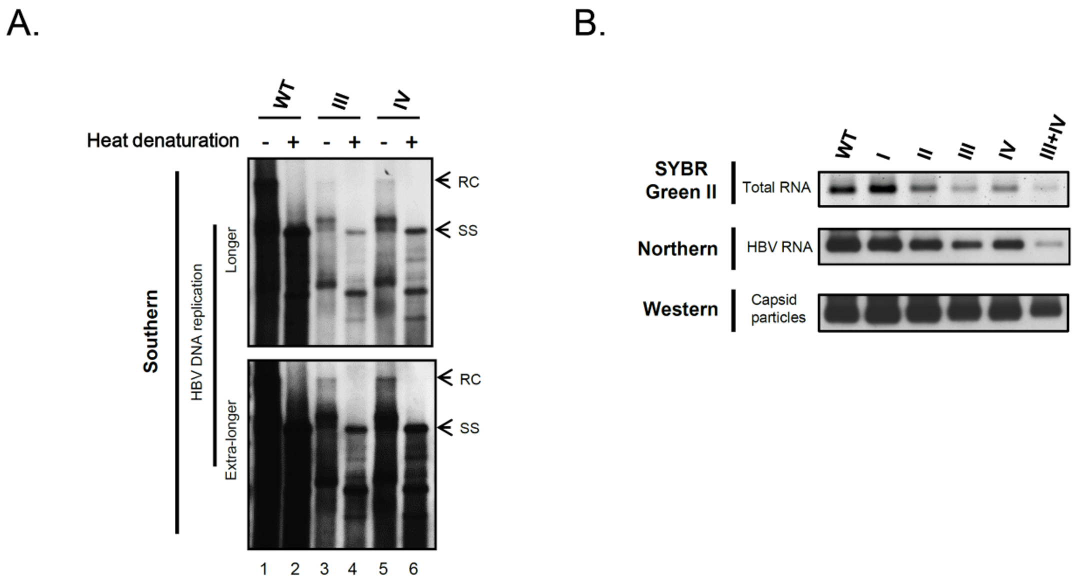

Figure S1. HBc mutants ARD-III and ARD-IV exhibited the most severe defect in HBV RNA encapsidation and DNA synthesis.

(A) By Southern blot analysis, full-length RC form DNA of single mutants ARD-III and ARD-IV can be visualized after extra-longer exposure of Fig. 1D. Heat denaturated (100°C, 5 min) viral DNA samples served as a size marker for SS DNA. RC: relaxed circle DNA; SS: single-strand DNA. **(B)** Consistent with the results in Fig. 1F, R-to-A mutations at different ARD subdomains revealed the differential position effects of arginine deficiency on RNA encapsidation as assayed by SYBR Green II staining (*upper panel*) and Northern blot (*middle panel*). These single or double ARD mutants are competent in capsid assembly as assayed by native agarose gel and Western blot (*lower panel*).

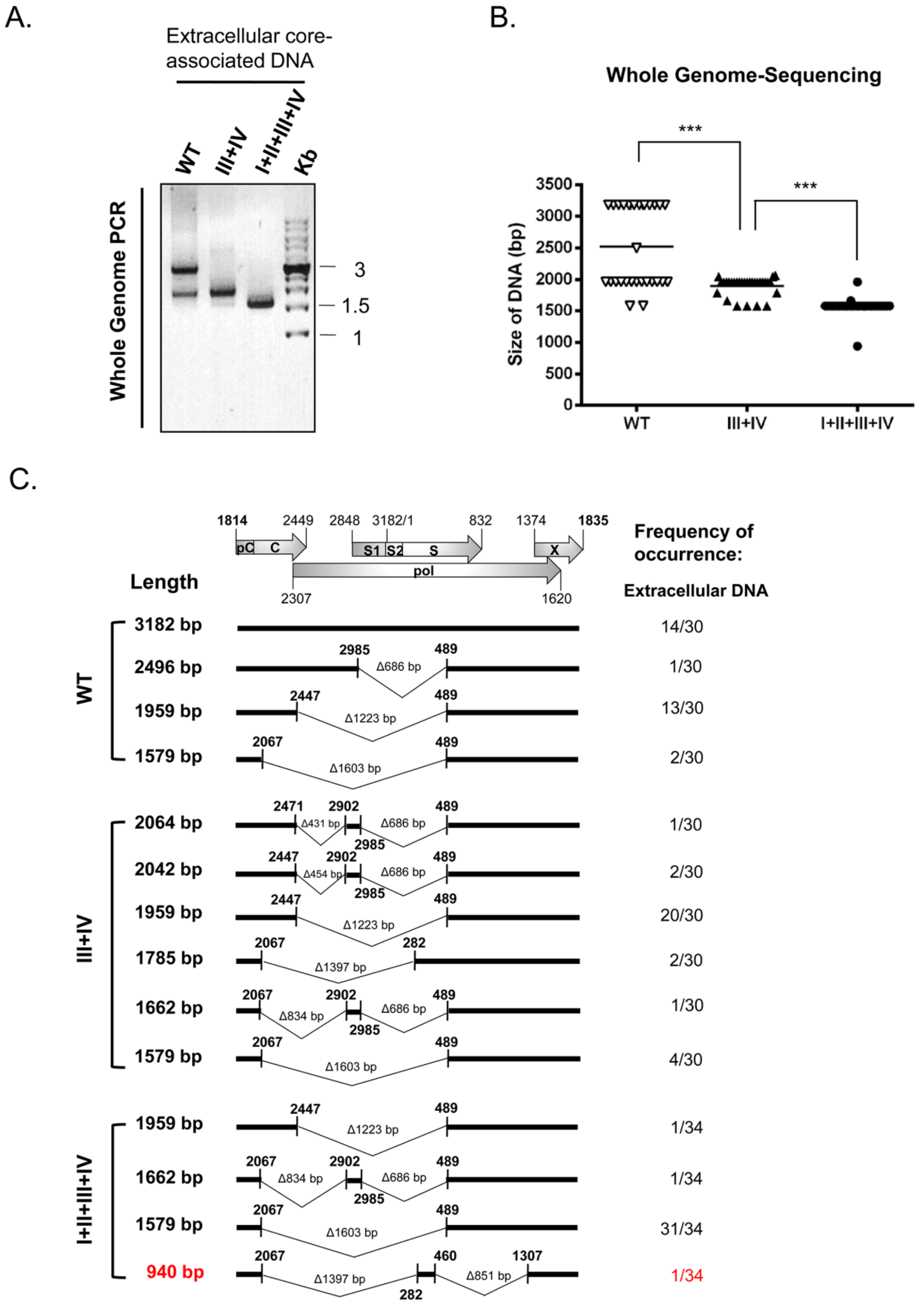

Figure S2. Secreted extracellular viral particles of arginine-deficient HBc mutants preferentially encapsidated spliced viral DNAs.

(A) Viral DNAs of extracellular particles were analyzed by Whole Genome PCR and

agarose gel with ethidium bromide staining. Compared to WT, mutant ARD-III+IV secreted shorter-sized of viral DNA, while mutant ARD-I+II+III+IV secreted the shortest-sized DNA. **(B)** The diagram summarizes the statistics of PCR sequencing results of extracellular particle-associated viral DNAs of WT, mutants ARD-III+IV and I+II+III+IV. Each symbol represents one independently isolated clone from *E. coli*. The horizontal line represents the medium size of secreted viral DNA species. ***$p < 0.001$, by Student's *t*-test. **(C)** This diagram is a summary of various frequencies of occurrence of each spliced DNA species in the extracellular particles in the media from HuH-7 cells transfected with wild type and arginine-deficient mutants. The red color represents a novel spliced DNA species not reported previously in literature.

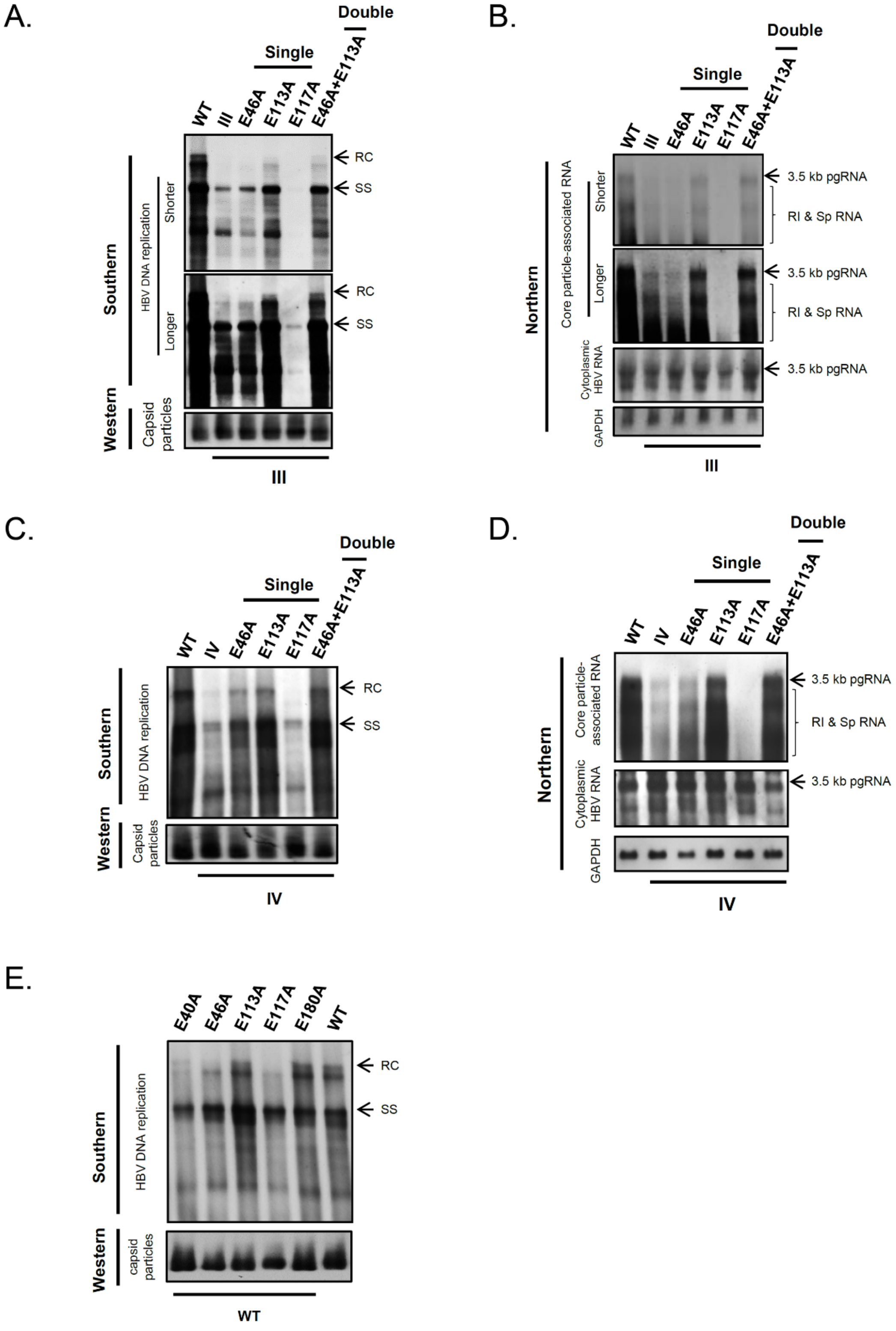

Figure S3. Efficient rescue of defective DNA replication and RNA encapsidation of HBc mutants ARD-III and ARD-IV by a charge rebalance approach.

(A) *Upper panel*: The defective DNA replication of HBc mutant ARD-III can be partially rescued by mutation E113A, but not by mutations E46A and E117A, by Southern blot analysis. *Lower panel*: Western blot analysis of intracellular capsid particles was included as a control. **(B)** The defective encapsidation of viral RNA of mutant ARD-III can be efficiently rescued by mutation E113A. Total cytoplasmic HBV RNA and GAPDH RNA were included as controls. RI and Sp RNA: replicative intermediates of reverse-transcribing viral RNAs and spliced RNAs. **(C)** The defective DNA replication of HBc mutant ARD-IV can be significantly rescued by mutation E46A, and fully rescued by mutation E113A by Southern blot analysis. **(D)** The defective encapsidation of viral RNA of mutant ARD-IV can be partially rescued by mutation E46A, and fully rescued by mutation E113A by Northern blot analysis. **(E)** Single mutations E40A, E46A, and E117A in wild type HBV context exhibited slightly reduced levels of RC DNA, while mutants E113A and E180A exhibited no significant change in viral DNA synthesis by Southern blot analysis. Western blot analysis of intracellular capsids on agarose gel was included as a control.

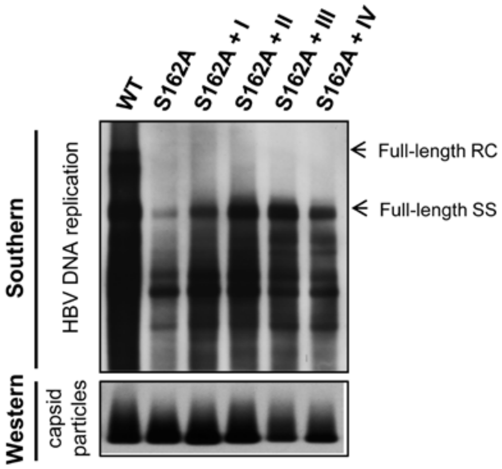

Figure S4. The replication defect of serine-deficient mutant S162A can be partially rescued by a second arginine-deficient mutation at ARD-I, ARD-II, ARD-III, or ARD-IV, respectively, by Southern blot analysis.

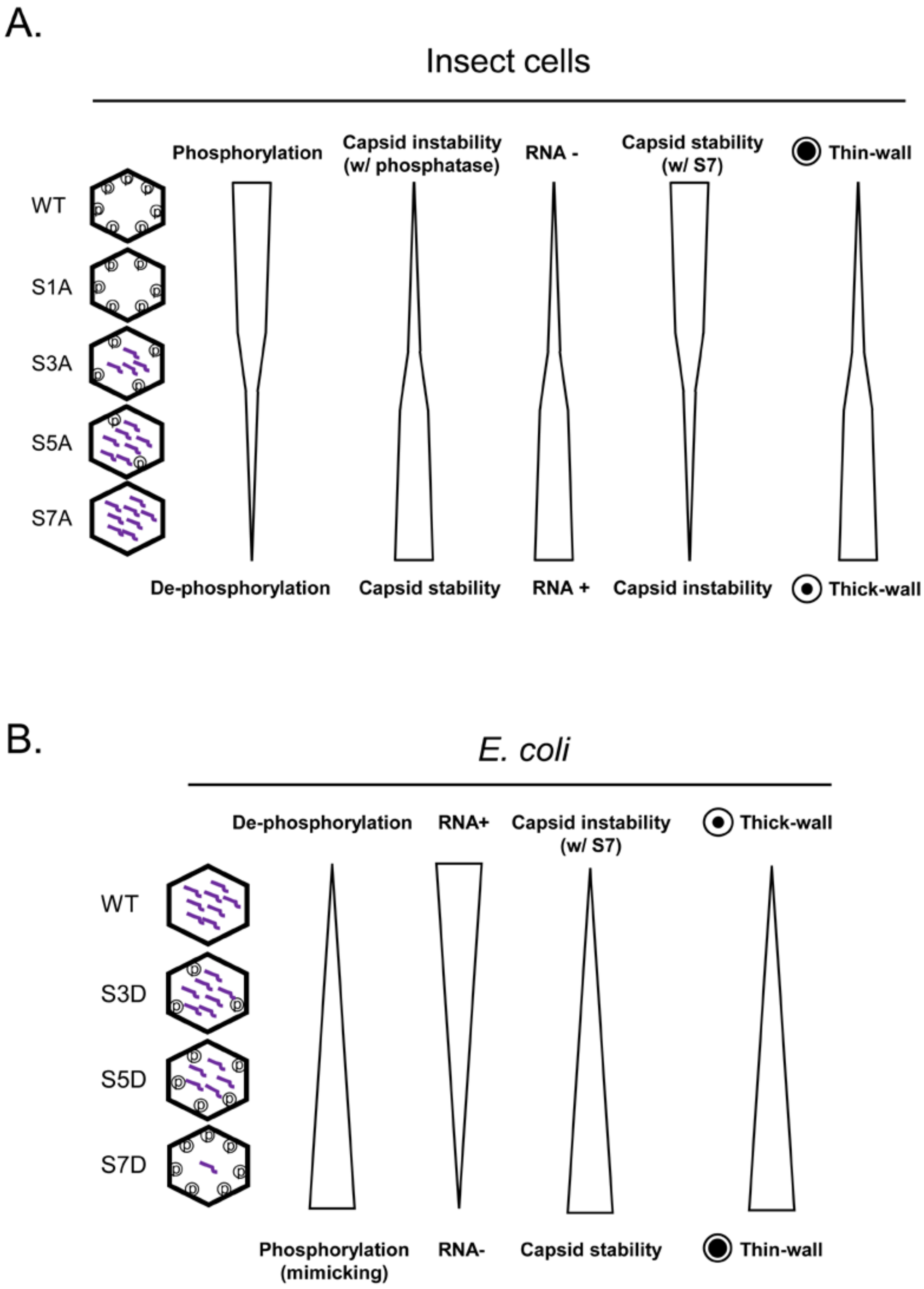

Figure S5. Cartoon illustrations of correlations among different virological and biochemical parameters of HBc VLPs prepared from *Baculovirus* and *E. coli* expression systems.

(A) A cartoon illustrates a general trend of correlations among several different parameters: 1) the degrees of serine de-phosphorylation of HBc ARD, 2) RNA encapsidation, 3) thick-wall capsid appearance, 4) capsid stability upon challenge with micrococcal nuclease S7 or 5) with phosphatase. **(B)** A cartoon illustrates a general trend of correlations between 1) the increasing content of aspartic acid (mimicking serine phosphorylation) of HBc ARD, 2) reducing amount of encapsidated RNA, 3) increasing resistance to micrococcal nuclease S7 treatment, and 4) increasing populations of thin-wall capsids from *E. coli*.

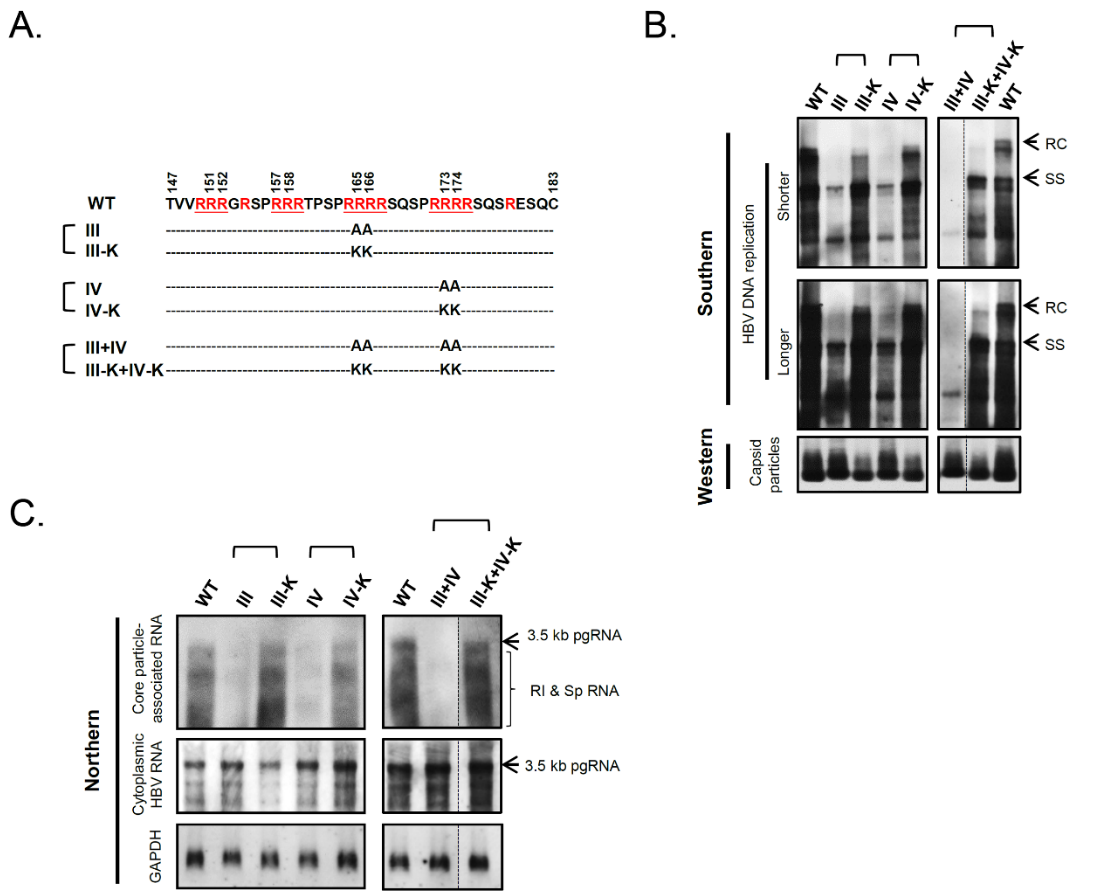

Figure S6. HBV RNA encapsidation and DNA synthesis depend on positively-charged residues of HBc ARD.

(A-C) HBV DNA replication and RNA encapsidation of R-to-A and R-to-K mutants at HBc ARD-III and -IV were analyzed by Southern and Northern blots. Unlike the R-to-A substitution, R-to-K substitution at HBc ARD exhibited less apparent phenotypic effect on viral RNA encapsidation and DNA synthesis. **(B)** In contrast to single R-to-K mutation, we noted that double mutations of R-to-K at both ARD-III and ARD-IV subdomains resulted in significant defect in RC DNA synthesis, albeit no appreciable defect in RNA encapsidation **(C).** The vertical dotted line indicates that

all lanes were from the same gel.
